# Fine-tuning the practical relevance of a quality framework for integrated nature-based interventions in healthcare facilities. A qualitative interview study

**DOI:** 10.3389/fpubh.2024.1379230

**Published:** 2024-06-05

**Authors:** Ann Sterckx, Ben Delbaere, Geert De Blust, Irina Spacova, Roeland Samson, Kris Van den Broeck, Roy Remmen, Hans Keune

**Affiliations:** ^1^Chair Care and the Natural Living Environment, Department of Primary and Interdisciplinary Care, Faculty of Medicine and Health Sciences, University of Antwerp, Antwerp, Belgium; ^2^Department of Bioscience Engineering, University of Antwerp, Antwerp, Belgium; ^3^Chair Public Mental Health, Department of Primary and Interdisciplinary Care, Faculty of Medicine and Health Sciences, University of Antwerp, Antwerp, Belgium; ^4^Department of Primary and Interdisciplinary Care, Faculty of Medicine and Health Sciences, University of Antwerp, Antwerp, Belgium

**Keywords:** transdisciplinary, co-design, quality assessment, nature-based intervention, one health, planetary health, healthcare, biodiversity

## Abstract

**Introduction:**

Integrated nature-based interventions in healthcare facilities are gaining importance as promising health and biodiversity promotion strategies. This type of interventions combines the restoration of biodiversity in the vicinity of the healthcare facility with guiding patients in that natural environment for health outcomes. However, quality appraisal of these interventions is still poorly developed. Based on a recent scoping review, the authors developed a preliminary quality framework in support of healthcare facilities designing, implementing and evaluating integrated nature-based interventions. This present study aims to fine-tune the practical relevance of the quality framework within the emerging practice.

**Methods:**

A qualitative interview study was conducted in seven healthcare facilities in Belgium. Using a combination of snowball and purposive sampling, 22 professionals, involved in the integrated nature-based intervention in their facility, participated in the study. The semi-structured interviews were transcribed and imported into NVivo. A deductive and inductive thematic analysis was used to explore the practical relevance of the quality framework. A stakeholders’ assembly review and a member checking of the findings were also part of the study.

**Findings:**

Twenty-two interviews with nature management coordinators, healthcare professionals, and healthcare managers were conducted by three principal investigators in seven healthcare facilities implementing integrated nature-based interventions. The contextualization and complexity of integrated nature-based interventions in the participating healthcare facilities demonstrated the need for an evidence-based quality framework describing nature-based interventions. The study led to nine quality criteria, confirming the eight quality criteria derived from a previous scoping review, and the identification of a new quality criterion ‘Capacity building, leverage and continuity’. These quality criteria have been refined. Finally, a proposal for a quality framework was developed and operationalized in a checklist. Deployment of the quality framework should be embedded in a continuous cyclical, adaptive process of monitoring and adjusting based on evaluations at each phase of an integrated nature-based intervention.

**Discussion:**

Bridging the domains of healthcare and nature management in the context of an integrated nature-based intervention in a healthcare facility requires a transdisciplinary approach. Scientific frameworks such as “complex interventions,” Planetary Health and One Health can support the co-design, implementation and evaluation of integrated nature-based interventions within a cyclical, adaptive process. In addition, the importance of the quality of the interactions with nature could gain from more sophisticated attention. Finally, the implications for healthcare facilities, policymakers and education are discussed, as well as the strengths and limitations of the study.

## 1 Introduction

Climate change and biodiversity loss require professionals and organizations to adopt a more sustainable, healthy relationship with nature (1–3), while recognizing the interdependent human-nature-health link (2, 4, 5). Consequently, supported by the Sustainable Development Goals of the United Nations, multiple professional sectors (e.g., energy, building, education, healthcare) and international academic and professional actors also respond to these challenges (4, 6). Examples of such actors are the One Health High-Level Expert Panel,^1^ the Planetary Health Alliance,^2^ and the Intergovernmental Science-Policy Platform on Biodiversity and Ecosystem Services (7).

Furthermore, ample evidence shows that exposure to nature can positively affect mental, social and physical health (8–14). In addition, scientific research emphasizes the value of developing nature connectedness (15–20) to benefit human and nature health (7, 21, 22). In that light, numerous studies have explored nature-based interventions (NBIs) to improve health and well-being (23–27). With this in mind, numerous healthcare facilities (HCFs) in Flanders (Belgium) are showing increasing interest in applying NBIs as a promising health promotion strategy for patients, careers and their staff. NBIs are ‘planned intentional activities to promote optimal functioning, health and well-being of individuals or to allow restoration and recovery through exposure to or interaction with authentic nature or technological nature’ (24).

Then, in response to the social-ecological crises, and in the light of sustainable healthcare, HCFs in Flanders also show interest in the topic of biodiversity restoration in the vicinity of their HCF. Moreover, evidence of the positive links between biodiversity and health (28–31) also fuels this interest. This common interest within an HCF brings nature management (NM) and healthcare together in the design and implementation, what we refer to as ‘integrated nature-based interventions’ (iNBIs). However, iNBIs in HCFs are a novel area in both research and practical implementation (32). HCPs require evidence-based practices to ensure quality care delivery. iNBIs in HCFs are complex interventions (33) due to contextual variety, diverse NBIs, and biodiversity restoration. Complex intervention frameworks, including Intervention Mapping (34) and implementation science (35), serve as the basis for their design, implementation, and evaluation. However, their use in iNBIs within HCFs appears to be limited (32).

Quality criteria play a crucial role in ensuring the effectiveness of interventions by supporting their design, implementation, and evaluation. They guide evidence-based, efficient, and tailored intervention development and ensure compliance with standards and best practices. Quality criteria also facilitate rigorous evaluation and identify areas for improvement. However, it is unclear which criteria HCFs use for iNBIs (32). Additionally, today, iNBIs do not fit straightforwardly into the prevailing frameworks regarding evidence-based practice and quality of healthcare. This is due to their versatility and complexity, but especially also because of their novelty. The existing healthcare quality indicators in Belgium^3^ (36) do not tackle the nuances and benefits of nature-based interventions. There is a gap in understanding and recognizing of the unique aspects and outcomes of incorporating nature and biodiversity into healthcare practices. Relying solely on current quality indicators may overlook important considerations essential for the successful design and implementation of iNBIs in healthcare. There is a need for a scientific and practice-based quality framework, tailored to the specific aspects of iNBIs, comprising a set of quality criteria, to facilitate the design, implementation, evaluation, and continuous adjustment of these interventions.

By addressing this gap, an evidence-based quality framework can support HCFs in a consistent and reliable implementation process of INBIs, their effectiveness and impact evaluation, while identifying areas, for improvement or adjustment (34, 37).

**Figure 1:**
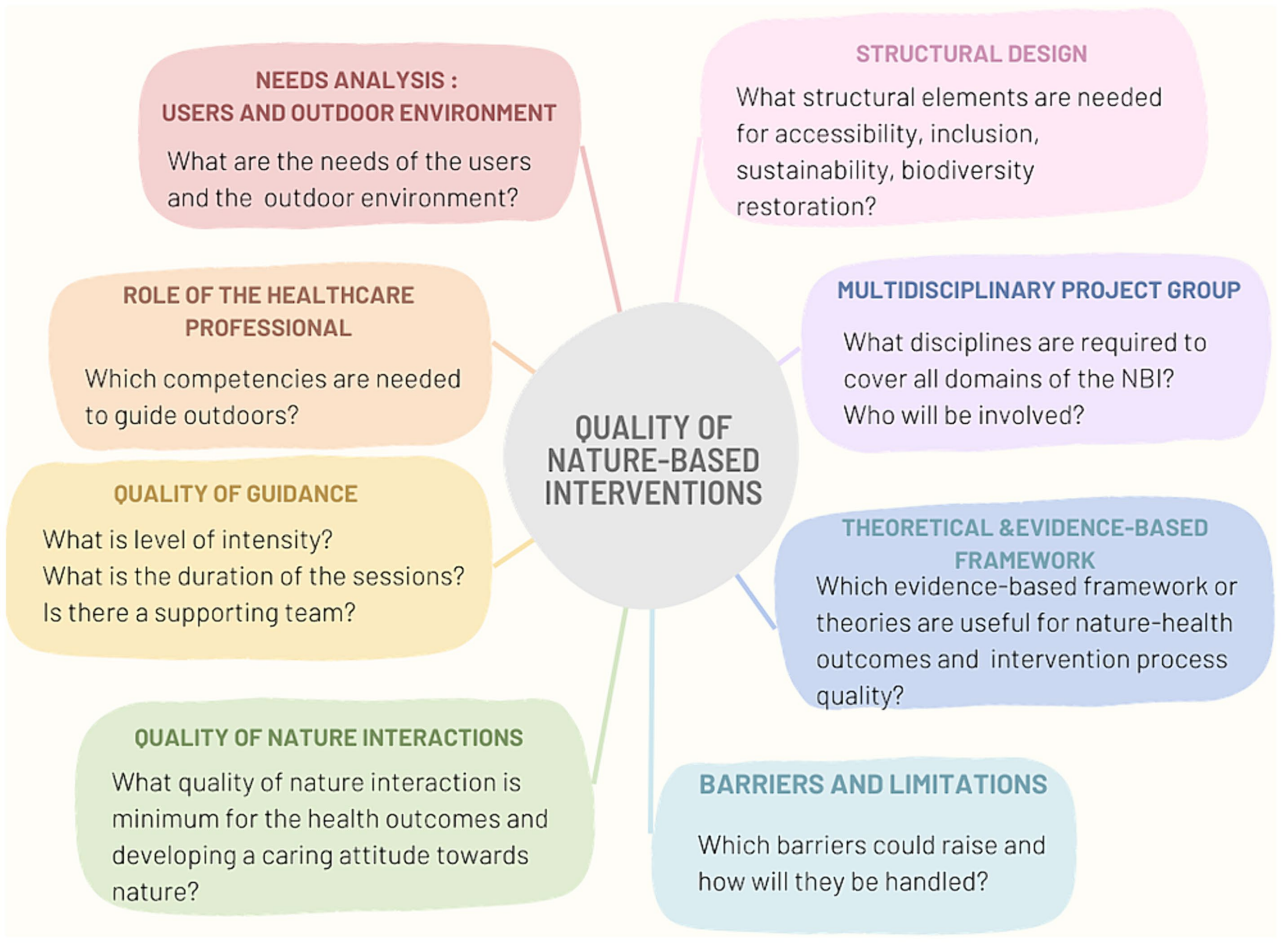
Preliminary framework with quality criteria for nature-based interventions in healthcare facilities. Reprinted with permission from (32), licensed under CC BY 4.0, https://doi.org/10.3389/fpubh.2023.1327108.

As part of a larger study, a scoping review (32) was conducted by the same authors on the quality criteria of NBIs in HCFs, which resulted in a preliminary framework. A visual overview of the preliminary set of eight quality criteria is given in Figure 1. In this study we further build on this preliminary framework to fine-tune its practical relevance.

To this end, we conducted a qualitative interview study with professionals in seven HCFs in Flanders, Belgium.

Our primary research questions in this study were ‘*Which quality criteria are used to design, implement and evaluate integrated nature-based interventions in healthcare facilities?’* and *‘How can we fine-tune the practical relevance of the quality framework?*’

## 2 Methods

**Figure 2:**
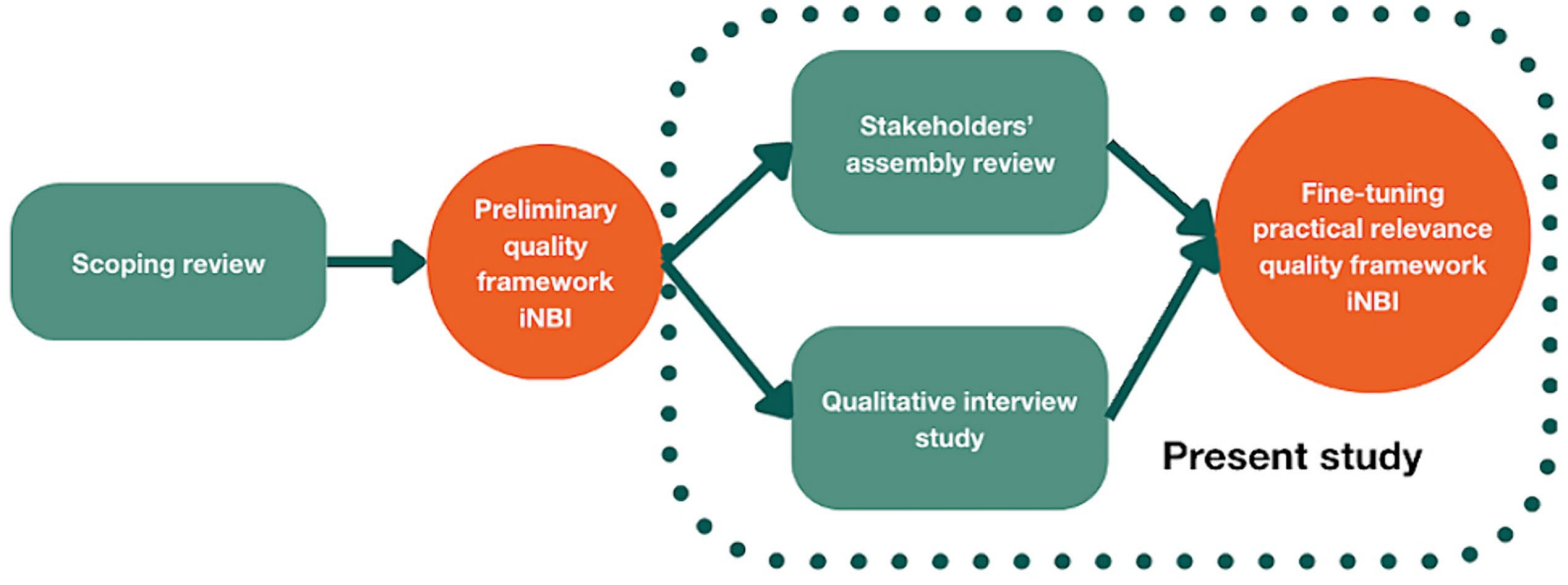
Multi-method qualitative study. The box contains the present study.

As part of a larger multi-method study (Figure 2) we first conducted a SCR (32) to identify the quality criteria underlying NBIs in HCFs. A preliminary quality framework was proposed (32). In this present study, we report a qualitative interview study with professionals involved in an iNBI in their facility, followed by a member check. In addition, data of a stakeholders’ assembly review was used as well.

The research team for this study consisted of an interdisciplinary research team, with experts in ecology, nursing, medicine, social science, micro-biology and psychology. This team collaborated closely with representatives from the Flemish Agency for Nature and Forests (FANF), a governmental nature conservation organization, and a representative from the knowledge center of the Flemish Infrastructure Fund for Person Related Matters, part of the Department of Care of the Flemish Government.

### 2.1 Respondents

Below we discuss the recruitment process of the respondents for the interviews and for the stakeholders’ assembly review.

#### 2.1.1 Respondents for the interviews

To ensure involvement of a diverse group of professionals across different HCF contexts, the recruitment procedure consisted of two steps.

First, for the recruitment of the HCFs, we used a FANF’s list of HCFs in Belgium (38), that had received funding for biodiversity projects since 2018. We conducted a purposive sampling with maximum variation (39) of HCFs based on the following criteria: focus on biodiversity and guidance of target groups in the surrounding natural environment within the same HCF; geographical distribution over the Flemish provinces (Antwerp, East and West Flanders); and several types of HCFs.The selection of the cases took place by the project group, consisting of the researcher team, the Flemish Agency of Nature and Forest (FANF) and the Vlaams Infrastructuurfonds voor Persoonsgebonden Aangelegenheden, department of the government Well-being, Public Health and Family (VIPA). A list of HCFs, funded for biodiversity projects at their HCF, was provided by FANF. In a first-round potential cases were discussed based on the selection criteria. The principal investigators moderated the meeting. FANF and VIPA created a ranking from one to 10, with one being the most appropriate in terms of relevance for the study and inclusion criteria. To account for the bias, AS and BD, two PIs, also prepared separate ranking and compared it with the ranking of FANF and VIPA. The average of both rankings was then calculated. The first seven cases (limited to number due to limited time frame and resources) were selected. Using this method, the selected cases consisted of five cases proposed by FANF and VIPA, and two cases selected by the research team. This step resulted in the selection of 7 HCFs, including 3 hospitals, 1 psychiatric hospital, 2 nursing homes for older people and 1 HCF for children and young adolescents.

Second, for the recruitment of the respondents a combination of snowball and purposive sampling (39) was used to recruit respondents in these HCFs. To begin with, snowball sampling was conducted, starting with the contact person of FANF in each HCF. They were asked who, being involved in the iNBI, would be willing to participate in an interview. Then, purposive sampling (39) was carried out based on the selection criterion of occupation. The sample had to consist ideally of at least one healthcare manager, one HCP, and one nature management coordinator (NMC), per HCF, to cover the several organizational layers involved in an iNBI.

### 2.2 Stakeholders’ assembly review

The stakeholders’ assembly was held to provide feedback on and refine the preliminary quality framework resulting from the SCR (32). Invitations to participate in the assembly were disseminated via email through a predetermined network established by the project group. The selected organizations encompassed a diverse range of sectors, including nature conservation and human health governmental entities, healthcare organizations, landscape architects, a human resources service partner, an organization that provides a patient platform, research centers of universities, as well as prevention and health insurance agencies. Of these organizations, a total of 22 representatives were selected. They were invited to disseminate the invitation for participation among interested potential stakeholders. Due to the necessity of promptly securing participants, convenience sampling methodology (40) was then used. Finally, the assembly comprised of five members of the project steering group and 11 potential external stakeholders consisting of two landscape architects, 2 HCPs, 1 researcher from a human resources service partner, 2 representatives of governmental nature organization, 1 hospital garden architect, 1 representative of a governmental health organization, 1 representative of health insurance organization, 1 representative of an external service for prevention and protection at work.

### 2.3 Data collection

#### 2.3.1 Interviews

After written consent, 22 semi-structured interviews were conducted with HCPs, healthcare managers and NMCs. The three principal investigators (PIs) (AS, BD and GDB) used an interview guide (Supplementary material) developed by AS and BD, based on the identified quality criteria from the SCR (32). Using the 5W1H method (who, what, when, where, why, how) guided the exploration of the previous identified criteria. This approach also left the possibility to identifying potential new criteria. The questions were divided into subsections, namely, (1) questions aimed at each respondent (e.g., the incentives for the iNBI, needs analysis, use of scientific evidence and frameworks), (2) specific questions for the HCPs and healthcare managers (e.g., guidance of the target group in the iNBI, their specific role in the iNBI, the nature interactions) and (3) for NMCs (e.g., the ecological quality, the design of the iNBI). Data collection took place between May and June 2023.

AS and BD conducted and recorded the interviews (approximately 1–1,5 h) with the HCPs. Field notes were taken during the interview. GDB conducted a walking interview (approximately 2 h) with each participating NMC in the biodiverse garden for practical reasons, including showing specific vegetation and landscape architecture. Extensive notes and quotes were written into a report and returned to the respondents for validation. The PIs had no previous working or research relationship with the participants.

#### 2.3.2 Stakeholders’ assembly review

The stakeholders’ assembly review was held in May 2023. First, a presentation on the preliminary framework, resulting from the SCR (32). Sixteen participants were divided into four groups to conduct a dialog for 20 min. Questions were shown on a flipchart (e.g., How do you experience this framework from the perspective of your expertise? What do you find positive and valuable about it, what about its applicability? What else would you find helpful in designing, implementing and evaluating an iNBI?). Finally, each group shared their feedback which was noted into a report by the PIs.

### 2.4 Data analysis

Using a social constructivist approach, we applied thematic analysis with a six-step framework (41). First, the PIs familiarized themselves with the data by transcribing the interviews, which afterwards were imported into NVivo, and pseudonymized. Second, deductive coding was performed based on the predefined criteria resulting from a former SCR (32), gathered into a coding scheme compiled by the two PIs. Next. inductive coding for identifying new criteria was conducted. Therefore, all interviews were coded separately by the PIs for a second time using a constant comparative method (42). The coding process continued until theoretical saturation was reached. Third, inter-coder reliability was assured by comparing the codes with each other. Only minor discrepancies occurred and were resolved by consensus (43). Fourth, the new codes were integrated into existent criteria or were merged into a new criterion. Fifth, the quality criteria were reviewed by the project group. Finally, the report was written.

Member-checking was performed by sending the summary of the findings to the interview respondents. Questions were asked about the refined quality criteria as a result from the interviews, and answers were offered as multiple-choice. The respondents had to check which option they recognized in their iNBI in the HCF (Supplementary material). In addition, the report based on the stakeholders’ assembly review was used to fine-tune the practical relevance of the framework.

### 2.5 Ethical approval

Ethical approval for this study was granted by the Ethical Committee University Hospital Antwerp with the number EC/Project ID 5395.

## 3 Findings

First, we give in 3.1. an overview of the respondents in the interviews. Second, we highlight in 3.2. the context of the participating HCFs with their characteristics and barriers regarding the domains of nature management (with focus on biodiversity) and healthcare (with focus on primarily NBI). Third, we discuss in 3.3. the quality criteria in detail, and separately to facilitate comprehension. Finally, in 3.4. we integrate the summary of the stakeholders’ assembly (3.4.1.) into the further development of the quality framework and a detailed checklist (3.4.2) operationalizing the quality criteria.

### 3.1 Respondents

A team of 3 PIs (AS, BD, GDB) conducted 22 semi-structured interviews, across the 7 HCFs with 5 healthcare managers, 9 HCPs (occupational therapists and psychiatric nurses), 6 NMCs, 1 quality manager responsible for NM and quality of healthcare, interviewed separately by two PIs (Table 1). One healthcare manager has been interviewed twice by 2 different PIs (GDB, BD). This person was responsible for quality of healthcare and for the nature management of the healthcare facility.

**Table 1:** Distribution of types of HCFs and respondents.

Code	Type of HCF	Target group of the iNBI	Number of interviews
HCF1	Hospital	Patients of 2 psychiatric units	3
HCF2	Hospital	Patients and staff	3
HCF3	Hospital	Patients and staff	4
HCF4	Psychiatric hospital	Patients and staff	3
HCF5	Nursing home	Older people	3
HCF6	Nursing home	Older people	3
HCF7	Youth care	Children and adolescents	3
			22 interviews

### 3.2 Context of the participating healthcare facilities

**Figure 3:**
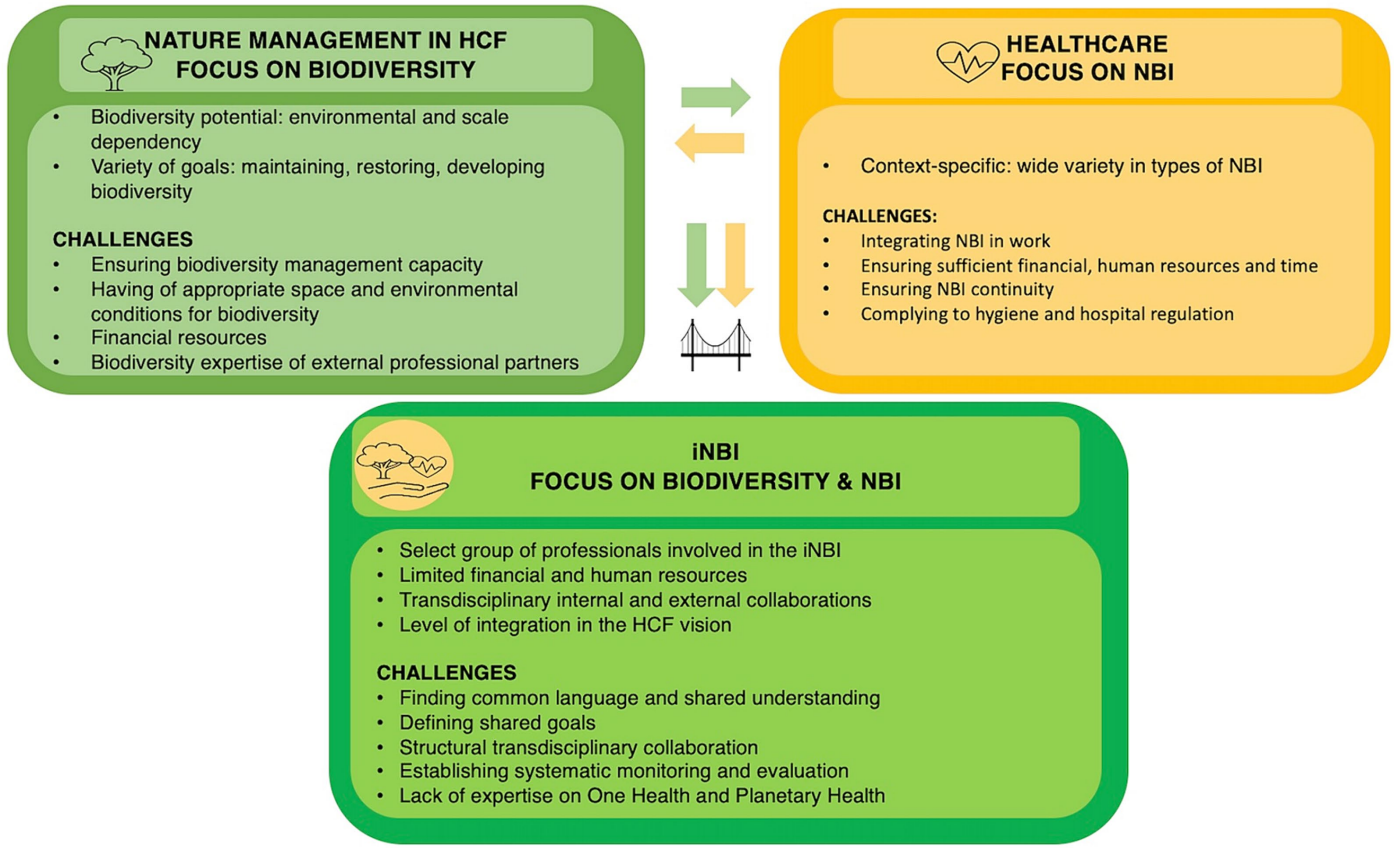
Contextualization of the participating healthcare facilities in this study with the domains of nature management and healthcare with each their characteristics and challenges, followed by their integration into an iNBI with its characteristics and its challenges.

In order to understand the findings on the quality criteria, it is important to first provide an insight into the context of the participating HCFs in relation to the iNBIs. Characteristics and challenges of the domains NM (with focus on biodiversity) and healthcare (with focus on primarily NBI) and their integration into the iNBI are briefly discussed. An overview is given in Figure 3.

#### 3.2.1 Nature management with focus on biodiversity

First, the responsibility for NM in the participating HCFs usually lies with the technical service or facility department, which takes care of the HCF facilities and the surrounding natural environment. Next, there is a difference among the HCFs concerning the scale of the surrounding natural environment and the type of nature (e.g., meadows, forest, pond), which affects their design and maintenance. The scale ranges from spots of a few hundred square meters scattered in the original HCF domain (e.g., HCF6) to a network of smaller and larger patches covering an HCF domain of several hectares (e.g., HCF3, HCF4). Some biodiverse pieces of land adjoin high nature-value land outside the HCF (e.g., HCF2, HCF4) that is managed and partially owned by a nature conservation organization. Others share this land, or pieces from this land, with the municipality (e.g., HCF3, HCF5).

In addition, the development potential of biodiversity depends on the environmental starting conditions. As a result, in our study one HCF may be at an early stage of biodiversity restoration (e.g., HCF5), while the other is already more developed and focused on maintenance within a master plan (e.g., HCF1). Furthermore, NM faces several challenges when integrating biodiversity into the NM of the HCF. First, it requires building internal nature and biodiversity management capacity. Sometimes HCF can rely on internal expertise (e.g., HCF1). However, mostly external expertise for biodiversity is necessary. Biodiversity requires appropriate spatial and environmental conditions, that are not always present from the start. Second, a biodiversity garden seems to require more financial resources, especially for its maintenance in the initial stage (mainly the first 3 years). Finally, depending on the context, the HCFs work with the NM department of the municipality or with commercial partners who may lack sufficient knowledge to effectively manage biodiverse vegetation.

#### 3.2.2 Healthcare with focus on nature-based interventions

When it comes to integrating NBIs in healthcare, there is a wide variety of NBI types among the participating HCFs, ranging from exposure to nature (e.g., sitting outdoors) to interaction with nature (e.g., sensory stimulation, gardening). For example, HCF5 currently limits its activity to nature walks, while HCF4 offers a variety of activities, such as walking, sensory stimulation, gardening, and animal care (e.g., chickens, goats, donkeys). However, NBIs are not always well structured in the tasks of HCPs, which threatens its continuity. Additionally, as the NBIs are conducted outdoors, they can sometimes conflict with hygiene and hospital regulations. For example, some pieces of land in which the NBI could take place are just outside the property of the HCF, resulting in potential issues of liability (e.g., HCF3, HCF6).

#### 3.2.3 The integrated nature-based intervention

When designing and implementing an iNBI, in which the two domains of NM and healthcare, initially functioning as silos, start to work together, new characteristics and related challenges arise. First, only a select group of professionals are involved in the development of iNBIs, with limited time, financial and human resources. Second, the extent to which biodiversity integration into the NM and healthcare practice is considered varies. For example, some HCFs have integrated the biodiversity-health intersection explicitly into their NM and healthcare vision (e.g., HCF1, HCF4). Others, although recognizing the attention for biodiversity at the HCF, have not explicitly taken into account biodiversity in their NBI (e.g., HCF2). Third, the iNBI involves a transdisciplinary approach, fostering internal and external collaborations, to discuss the shared and respective needs and goals of each field. However, this is often a challenge as it is influenced by the vision and culture of the HCF. Furthermore, there is a lack of knowledge about systematic monitoring and evaluating the iNBI. Finally, informing the iNBIs from a One Health and Planetary approach appears not to be common practice yet, mainly due to a lack of expertise in the HCF.

Overall, the contextualization of iNBIs in the participating HCFs demonstrated the need for an evidence-based iNBI quality framework. To ensure the quality and continuity of iNBIs, this framework should be context-specific and adaptive to address the respective and common needs and challenges of each domain. The fine-tuning of the practical relevance of the preliminary framework from the SCR (32) will be discussed in the following, which is divided into two parts. First, the refinement of the quality criteria based on the interviews is discussed in 3.3. Second, in 3.4. we will discuss the fine-tuning of the practical relevance of the quality framework.

### 3.3 Quality criteria of integrated nature-based interventions in healthcare facilities

The interviews led to the refinement of the eight quality criteria, previously identified in the SCR (32), and the identification of one additional quality criterion ‘Capacity building, leverage and continuity’. The quality criteria will be described based on their operationalization in practice.

#### 3.3.1 Quality criteria regarding the intervention process in the integrated nature-based intervention design and implementation

This quality criterion has been refined compared to the one identified in the SCR (32).

##### 3.3.1.1 Needs analysis of users, the outdoor and natural environment

The needs for healthcare are usually discussed in workgroups consisting of colleagues with relevant expertise on the target group, including a person-centered approach. For example, the needs for each target group vary (e.g., dementia patients versus patients with anxiety disorder), which increases complexity when the natural environment is used by different target groups. Aspects mentioned were outdoor accessibility, safety (e.g., only edible plants; presence of a pond; surface of the paths) and available activities (e.g., relaxation, activation, sensory activities). Other aspects were the necessary levels of guidance and the extent of control (e.g., the necessity for cameras outside, presence of an HCP outside to supervise), and appropriate plants for sensory experiences.

*“You must dress them (people with dementia) a bit more, put sunscreen on them anyway when the sun is shining strongly, so yes, there is a bit more work involved. You must also see if there are any residents who would run away, so you must make sure that someone is always with them. Also, people in wheelchairs, yes, they need someone to push them. ‘(HCF5 HCP).*

With regard to staff, respondents mentioned that they primarily need places in the natural environment to relax, retreat, or exercise (e.g., go for a walk). For example, at one HCF, the design team had decided to build three circuits according to the available time of the HCPs.

*“We have three circular walks, and we have been taking into account that our staff do not have that much of a break, so we said we should also foresee a short circular walking route.” (HCF2 healthcare manager).*

As a rule, the analysis of the ecological needs of the surrounding natural environment is left to the technical department. However, if there is ecological expertise in-house, clear decisions will be made in this regard. In other cases, an external expert is consulted when necessary. In one case, the vision for the design of a hospital campus is clearly formulated in a master plan: it should offer a ‘healthy place’ in its natural environment for various target groups.

*“Healthcare is central, with the central hospital in a “healing environment” where it is pleasant to live and work. The campus will be a healthy place where it is good to heal, work, study, and stay with plenty of space for nature, walking and cycling paths, and green meeting places …The vision of the hospital is clearly outlined in the ‘Environmental Standard’ in which typologies and guidelines to be followed are elaborated for the* var*ious elements that determine the quality of the campus environment for people and nature.” (HCF1 NMC).*

##### 3.3.1.2 Goal setting

Goal setting happens at the start of the iNBI project. In several HCFs, the goals of the iNBI design appear to be multi-focused. First, it involves creating relaxation spaces in a natural environment that allow natural experiences and improve the well-being of target groups. These spaces can encourage garden walks and involve family members in these activities. Second, the iNBI design aims to restore biodiversity around the HCF, often in combination with sensory experiences. Third, the iNBI should promote interaction between the HCF and the local community by sharing the natural environment of the HCF.

*“The main goal is often activation of going outside, but colleagues do also take the lead, for example, in a crisis situation, by taking a walk together outdoors or letting people go outside to recover from an intense conversation.” (HCF1 HCP).*

Finally, one HCF mentioned that their goal of the iNBI consisted of using the natural environment to have conversations with long-term absent staff. Conversations outside or during nature walks can feel more inviting and pleasant than conversations in the office.

*“Then we contacted our long-term absent staff because we wanted to keep in touch with them and noticed that it was difficult for them to come to the hospital. Especially because entering there becomes a barrier, which releases emotions. They do not want to see colleagues, that’s difficult. Then we thought, what if we use the forest and invite them for a nature walk.” (HCF4 healthcare manager).*

##### 3.3.1.3 Evaluating the process and impact of integrated nature-based interventions

Monitoring and evaluating the quality of the intervention process and of its impact is imperative for ensuring the quality of healthcare and ecological interventions.

Several HCPs mentioned the need for more systematic and scientific evaluation of the iNBI process, its impact and possible adverse outcomes on health. However, often HCFs lack the expertise and resources to evaluate the progress and impacts of the iNBIs.

Usually, HCPs rely on their personal evaluations and observations about the patients’ progress and discuss their experiences informally in follow-up meetings. The NMCs observe the changes and evaluate their compliance with the biodiversity objectives. Although its progress is usually discussed in workgroups, there is also a lack of formal structural monitoring of its maintenance and progression.

*“I think mainly spontaneous feedback, but also discussion about whether you were able to achieve your goals, which we also discuss at the end of the session. And then sometimes we also look ahead to next week. For example, when we guide people with fear of contamination, you can consider where there are still challenges and go one step further with them.” (HCF1 HCP).*

#### 3.3.2 The use of evidence-based frameworks and practices

Most HCFs conduct initial exploratory scientific literature research at the start of the iNBI design. Sometimes, an HCF establishes contacts with research experts, or gets involved in a research project or bachelor programs.

Relying on evidence-based practices seems crucial, especially in hospitals. However, given the context-specific nature of iNBIs, practical feasibility is paramount here.

*“When we start something new like this (an iNBI), it is important that someone has already tried it out and that we can rely on the literature. We always start with a literature review and then we look at what already exists, but when we get into practice, we also let go of that to some extent, like, okay the science is there but we look to what we can use practically and what is workable for us.” (HCF1 HCP).*

Some HCFs rely for biodiversity restoration on the scientific literature of appropriate methods and typologies. They also use knowledge of the local environment and its species and are mainly based on science-based ecosystem approaches.

*“This approach is characteristic of the integrative way of working that is maintained here. The environment, sustainability, and biodiversity are always considered together. Typologies regarding plants and vegetation’s and their role and application are based, among other things, on the required habitat factors, allergens that can be produced, fine dust that can be captured, but also on the intensity of management and maintenance they require, the space above and below ground they need for growth and development.” (HCF1 NMC).*

#### 3.3.3 Establishing transdisciplinary internal and external collaborations

The development of iNBIs requires to establish transdisciplinary internal and external collaborations in the design and during the iNBI implementation.

Internal collaborations are established between the technical department or the inhouse garden architect, occupational therapists and management representatives of human resources, quality and prevention.

*“…The management team, people from the technical department, an occupational therapist, the physiotherapist, and a patient care coordinator (...). (HCF 5 healthcare manager).*

An interesting example is an HCF where in the iNBI implementation a partnership was established between the occupational therapy and the technical department, to support each other’s needs. For example, occupational therapy may support with their target group the technical department with garden related tasks, such as raking leaves and bringing them to the garden.

*“... and we also have the great advantage that we have the technical service that maintains here the green space: they prune those trees, and then we have parcels of branches ready so that they (participating patients) just have to drive to the forest with the parcels. (...) We do try to do a lot for each other ... we want to get rid of the compartmentalization that used to exist, you cannot keep that up, but there must be someone who does the coordination” (HCF 3 HCP).*

External collaborations are also established, often involving volunteers in care (e.g., taking patients outside for a walk in the garden) and biodiversity (e.g., helping to maintain the garden). The municipality or governmental partners in nature conservation may also participate in joint biodiversity projects. Collaborations with other HCFs on the same site or in the surrounding area have been reported, for example, when they share the surrounding natural environment. Finally, collaborations with health associations for special care needs (e.g., association for informal care) were also mentioned (e.g., creating together a resting and mourning place in an adjacent forest).

#### 3.3.4 Capacity building, leverage and continuity of the implementation of the integrated nature-based intervention in the healthcare facility

This new identified quality criterion shows several indicators that can be used to evaluate the capacity building, leverage and continuity of the implementation of the iNBI within the HCF.

First, it relies on colleagues and management recognizing and supporting the use of iNBI and its dissemination within the HCF. Second, capacity building of iNBIs is reflected in the number of HCPs involved and the degree to which different levels of the organization (employees, management, and board) are involved in the design and implementation of iNBIs. Third, leverage is evident in conversations with patients about the iNBI, inquiries by any user related to the iNBI (e.g., outdoor activities) and ideas expressed by HCPs (e.g., insect hotel, flower picking garden). Fourthly, staff’s use of the natural environment for personal health is also a positive sign. Fifth, spontaneous, positive feedback from visitors or relatives shows leverage. Effective communication strategies, such as official garden openings, leaflets, posters, communication boards, residents’ newspapers, and internal news releases, play a crucial role in capacity building and leveraging.

Regarding communication challenges, an HCF faced adverse reactions to a recently established biodiversity garden that contradicted the traditional notions of a “clean hospital.” To address this issue, the HCF improved communication through notices in internal newspapers and communication boards (“This is an area undergoing maintenance”). Strategic design, management of biodiversity areas, and appropriate communication are crucial and must, where possible, be guided by the expectations of staff, patients and visitors, mainly when introducing ‘rewilding’ or enabling places with spontaneous nature. Sometimes zoning strategies are initially used in garden design or developed gradually to manage green spaces effectively, where people can adapt to the new image of biodiversity.

*“When I think about functions, users, and desirable nature management, I see three levels. The macro level is the general healing environment of the campus for everyone, with the zoning of spontaneously dynamic nature in the edges and more controlled nature closer to busy buildings. A visitor, but especially the staff, gets to those edges. The meso level is linked to specific departments of the HCF,* e.g.*, rehabilitation or anxiety and mood disorders. Here, the design and management of outdoor and indoor green spaces are entirely dedicated to the care of the target group. Finally, the micro-level relates to the very personal functions of a person, such as mobility or sight impairment, and how greenery and nature can improve the quality and experience of these. Think, for example, of “guidelines or signposts” for the visually impaired that allow that the space can be experienced in multiple ways.” (HCF1 NMC).*

Continuity of the implementation of iNBIs turns out to be a sensitive issue, especially when there is high staff turnover or long-term absenteeism. Factors such as budget constraints or negative perceptions of biodiversity gardens among residents or staff can jeopardize the continuity of the iNBI implementation. For example, a respondent who worked in a nursing home testified that they readjusted their lawn care policies from “allowing to grow wild” to regular mowing, when the residents complained about “bad weeds” and that “tall grass” is a sign of an untidy lawn.

In all, ensuring continuity and resilience of the iNBI implementation to disruptions as mentioned above, depends on embedding iNBIs in the overall HCF vision and policies of the organization.

#### 3.3.5 Structural design

The structural design of iNBIs is crucial and has variations tailored to their ecological context and target group. They vary in scale, from focused areas addressing specific care needs to entire HCF domains transformed into holistic healing environments. These initiatives can arise from previously barren areas or evolve from existing gardens, green spaces, or forests. The longevity and evolution of an iNBI have significant implications for its ecological functionality, distinguishing between newly constructed projects and long-standing green environments. The design can range from various green elements, from forests, parklands, and landscaped gardens with herbaceous vegetation to animal husbandry areas, open water areas, or picking meadows.

For patients, visitors and, occasionally, the local community members, access to natural environments is a major concern. For example, some HCPs have facilitated the use of wheelchair outdoors, adapted garden paths for wheelchair users, or created paths that allow easy access to nearby forests.

*“We have also had three additional vegetable garden troughs put up with a raised pedestal where people with wheelchairs can also pass under them, and as such the garden was adapted to all kind of people.” (HCF6 healthcare manager).*

In addition, considering sustainability in the design and material selection appeared to be crucial for the iNBI design. For example, the focus can be on the ability of the property to retain and permeate water. Implementing semi-paved surfaces, such as roads, wadis, and partially open ditches, is adaptable to different environmental conditions. Furthermore, integrating environmental sustainability and biodiversity should be consistent with healthcare requirements. For example, an internal document within an HCF provides detailed insights into elements that determine the quality of the environment for people and nature and provides various typologies and implementation guidelines.

*“For example, the 16 to 17 typologies of paving (if it is necessary anyway) with the different tiles or materials that can be used for that purpose. Apart from their carrying capacity, they differ from each other in the degree to which infiltration is possible or not in their own beds, the vegetation that can develop in them, but also in the degree to which fine dust is released, that they cause more or less warming, in the impact of their production on the environment and climate.” (HCF1 NMC).*

Structural elements such as barefoot paths, benches, exercise equipment, playgrounds, and auditory elements may be considered when designing gardens or natural spaces. These elements serve various purposes, including providing resting places during walks and encouraging the patient’s engagement with nature. In addition, they facilitate the participation of a wide range of users, including children, visitors, and the local community, thereby promoting interaction between the HCF and its neighborhood. Although these elements may not be specifically aimed at improving biodiversity, certain additions such as insect hotels, beehives, nesting boxes, growing native plants, and selecting seed mixes and perennials can positively impact biodiversity. They can also enhance sensory experiences at the same time. In addition, signs that indicate routes or rest areas make it easier for users to navigate and feel comfortable in these environments.

*“We also collaborated with a wood sculptor who created really nice rest areas, because we also noticed that patients who visit these areas, have walking difficulties, and want to rest a bit.” (HCF2 healthcare manager).*

#### 3.3.6 Role of the professionals involved in integrated nature-based interventions

The role of the professional involved in the iNBI was highlighted by the respondents by the need for specific competencies and by their personal relationship with nature when dealing with iNBIs.

##### 3.3.6.1 Specific competencies needed when an iNBI is implemented

All HCPs in this study demonstrate pioneering characteristics by daring to experiment while maintaining a strong focus on the needs and safety of their target groups. Furthermore, being involved in an iNBI requires specific competencies. First, they need to develop flexibility to adjust their responses to unexpected and complex circumstances, such as weather and ecological conditions, the patient’s perception and feeling about going into nature, and the specificity and care needs of the target group when guiding in a natural environment.

*“As a therapist, I also feel like I must be very flexible and that there are a lot of unpredictable things that come my way, if you have in mind that ‘I am going to do that this afternoon, always something else can happen’. That is true in nature; the weather can be too bad to work outside. It may be that someone is too tired or feels very bad and does not want to do that work. It can be too hot, too dry, too wet. It may be that the plants may not have germinated yet or that they are not successful, and you have to sow again and follow the rhythm of nature and of the patient.” (HCF4 HCP).*

Second, during the implementation, HCPs need to learn by experience. First, most HCPs look up information about evidence-based practices and other information (e.g., ecological, gardening), but gradually, they rely on their experience, and adjust as the iNBI evolves.

*“We try it our own way and learn from each other what we like, what we think is good and, above all, what’s good for everyone. We went on really nice walks in the snow, spent an entire afternoon in the woods, and were able to show things to each other. Things we heard, smelled, and felt. So, yes, go to read about it, and then learn about it yourself.” (HCF4 HCP).*

Third, developing ecological awareness, such as conserving the ecological quality and inspiring their target group with ecological knowledge were mentioned as well.

*“I always saw him (a famer with tractor) racing out of my window, and then I called my colleague (from the technical service), and said: ‘Y, that is not how we like it; tell him he should drive more slowly, because that is not good for the soil’. That was all explained to me by a nature conservation organization, the way you mow something and what you do with that grass. And then Y said, a few months ago, ‘it has been successful, there is a new tender, and it will be someone who is more in line with our vision, and then I thought, OK, because that is assurance.” (HCF3 healthcare manager).*

Finally, openly sharing their personal nature experiences, also known as self-disclosure, can increase the support and leverage of iNBIs among colleagues, patients, and other potential users.

*“Yes, I can talk a lot with residents about farm life because many have such a background, and yes, that is something they are less able to discuss with other colleagues about what it was like in the past and what it is like now. We also occasionally go on trips to a farm, and so on.” (HCF5 HCP).*

The NMCs usually have specific expertise in garden or landscape architecture, nature or forest management, or ecology. The NMCs also learns from external experts (with whom they temporarily work together). However, they experience that it is crucial to manage potential conflicts between healthcare needs and those related to biodiversity. To effectively manage these responsibilities and tasks, they therefore require competencies similar to those seen in HCPs. For example, a flexible attitude is required to deal with challenging situations. Such challenges can be loss of newly planted vegetation due to sudden climate changes, or when biodiversity restoration initially requires more time and expertise to maintain. Additionally, challenges may arise when HCPs, residents or visitors do not understand why certain pieces of land are left to spontaneous ecological processes.

*“It is clear that using a garden for therapy makes a lot of sense, but that the garden should then look as it does now (maintenance according to biodiversity guidelines) is not clear to everyone.” (NMC HCF5).*

##### 3.3.6.2 Personal relationship with nature

Interestingly, all respondents expressed how their relationship with nature motivated their participation in designing and implementing iNBIs in their HCF. The HCPs engage with nature in various ways, from outdoor activities such as walking or jogging to caring for their gardens and animals at home. Additionally, they enjoy observing and appreciating the cyclical patterns of the seasons and finding symbolic meanings in what nature offers, which demonstrate a sense of nature connectedness. Furthermore, nature provides them a sense of tranquility and a break from stressful work experiences. Others find happiness and beauty in nature and feel a sense of awe that fuels their desire to engage with it.

*“For myself, I have also been there (in nature) a few times to unwind, you are always between those four walls here. But in the afternoon when the weather is good, yes, even though there is a big building here, you really have the feeling that you are ‘in’ nature in that forest.” (HCF2 HCP).*

#### 3.3.7 Quality of the guidance in integrated nature-based interventions

The quality of the guidance is determined by the nature of the profession, which allows to easily take on the guidance of iNBIs and apply person-centered care in the design and implementation of INBIs.

Most occupational therapists, physiotherapists, animators, and other HCPs are involved in guiding their target group within iNBIs. Regarding the occupational therapists, the peculiarity of their occupational practices allows them to integrate nature seamlessly into their work. In addition, some occupational therapists work across departments, allowing for greater awareness and easy dissemination of iNBIs throughout the HCF.

*“My colleagues are also involved; if it (the NBI) was limited to the healthcare department only, it would be more difficult, but I think the physiotherapist and occupational therapist together do their best to make that possible.” (HCF5 HCP).*

The importance of person-centered care, where iNBIs are tailored to users’ needs, has been previously highlighted in the SCR (32). However, elements underlying a person-centered approach, such as intensity, frequency, and duration of the sessions, were not explicitly mentioned by the respondents in this study. Nonetheless, it became clear that complexities arise when iNBIs serve multiple target groups simultaneously (e.g., shared use of a biodiverse garden) or when different disease types are present within a single group (e.g., in an open program). Conflicting interests can therefore impact iNBI implementation, necessitating the need for multiple supervisors and decisions on structural design and vegetation selection. While certain elements may benefit one person, they may pose risks to another, creating unintended outcomes and challenges in achieving a balance that safely and effectively meets different needs.

*“For people with anxiety disorder who find it hard to go outside, yes, then that will be the objective for them. First on the terrace and then always a little step further. For someone with a compulsive disorder, exposure to soil, when someone is very anxious about excrement, then soil can feel very dirty. So, trying to enter that vegetable garden can be a goal in itself. And for someone with a depressive state, it is mainly about acting and having success experiences and feeling like you have done something meaningful. So, I think it can be meaningful for everyone, but then it is up to you, yes, how do I apply that? (…) And as an occupational therapist we then look at the individual patient with their individual objectives.” (HCF1 HCP).*

#### 3.3.8 The quality of the interaction with nature

Our study confirmed, as already stated in the SCR (32), that iNBIs contain different qualities of interaction with nature. The choice of the quality of nature interaction is based on the HCP’s experience and knowledge of its target group. Table 2 gives on overview of the different qualities, ranging from activities in nature to sense of purpose, with their description and an illustrative quote.

**Table 2:** Overview of the different qualities of the interactions with nature.

Type of interaction	Description	Quote
Activities in nature	The most common nature interaction is activities in nature, such as walking with patients in the garden or in the natural environment of the HCF	*“Two permanent colleagues take patients on a walk, patients that we can trust, there is a lot of cooperation there.” (HCF1 HCP)*
Gardening	Gardening is a common activity in various HCFs. Being in nature and gardening is a suitable medium for people who can work less quickly, also because the rhythm of nature can be followed.	“*Gardening is actually a lot of watching, seeing what happens, what should not happen, what is desirable, where should I intervene, where should I not intervene... It is also a lot of waiting, waiting for that seed to come out, for the leaves to grow. Arriving... waiting for tomatoes to arrive... and that rhythm is actually almost tailor-made for people who are no longer productive, so to speak. Also, the rhythm of what nature does, that is a constant interaction and adjustment*.” (HCF1 HCP)
Sensory stimulation	Sensory stimulation is reported in various HCFs. For example, perception training is used in which taste is stimulated and smells and structures are used. A biodiverse and experiential garden is perceived as supportive due to the multitude of natural stimuli, and thus a rewarding environment for stimulating sensory experience.	“*This is a garden that stimulates the senses. Encourage young and old to actively participate. We use natural elements such as trees, shrubs, herbs, tactile and movement elements... For people with dementia, sensory perception is often the only way to experience. This way they become aware of the world around them.*” (HCF6 HCP)
Aesthetical experiences and beauty of nature	Patients are also taken to nature for enjoying aesthetical experiences and admire the beauty of nature.	“*I gave that group of older people an hour of nature. It was not just a vegetable garden but various things on the property; admire what was there at that time. In the spring the magnolia blossoms or other things that people do not always know about that they are on the property. There was always a lot of response and gratitude to be there.”* (HCF4 HCP)
Nature as a teacher	Nature is sometimes used as a teacher, a mirror, for example as a metaphorical reflection that nature or certain ecological processes provide. Metaphors are expressed not only by saying them, but also by physically experiencing them, such as in gardening (e.g., planting a seed and taking care during its growth).	*“For example, we choose yin yang beans, not so much to eat, but those are beans that have a white and a black part. With a white dot on the black part and a black dot on the white part. Then I ask, is there anything you can do with that? What do you experience with that symbolism? In addition to the good there is also the difficult or the bad or in addition to the bad there is also the good and grab it when you are having a hard time.”* (HCF4 HCP)
Healing power of nature	Nature is also often used due to the healing power it offers. Examples of this are losing track of time, forgetting worries, offering comfort in moments of grief. For employees, healing is mainly aimed at being able to relax, withdraw for a while, or recharge their batteries during short moments or breaks.	*“The pastoral service once asked the patients ‘What helps you?’. They replied ‘enjoy nature and the environment, it is healing’. (…) I thought about that group that goes on a walk with psychotic people, the effect of knowing that you are connected to nature can be so healing.”* (HCF4 HCP)
Sense of purpose	Gardening or helping to care for the natural environment gives some patients a sense of purpose, and the feeling to experience the life cycles (for example, the cycles of the seasons, watching something grow).	*“This morning there was also a gentleman who was in a bad mood and after he had watered zucchini he said that he was satisfied because he had done something meaningful. While in a simulated setting where you sometimes, yes, I am also an art therapist, work with all kinds of material, you sometimes have less questions about ‘what am I doing here’, even though that is also meaningful.”* (HCF1 HCP).

#### 3.3.9 Contextual conditions and obstacles

Various constraints and obstacles encountered in the implementation and iNBI continuity were reported.

##### 3.3.9.1 Lack of time and staff

The lack of time poses constraints for taking patients and staff outside, as their breaks are frequently short. On the other hand, proximity to nature makes it easier for staff to take breaks outdoors or have lunch. Additionally, staff shortages or prolonged absences can jeopardize the continuity of the implementation of iNBI initiatives (e.g., maintaining flower garden), causing such projects to be halted during such periods.

*“Now, at the moment, in the healthcare sector, it is not always convenient to have enough hands at the bedside; we also notice that healthcare providers make less use of it (going into nature).” (HCF5 HCP).*

The NMCs mentioned lack of time, resources, and staff turnover as key obstacles. Staff turnover contributes to losing expertise and knowledge and potentially affects the continuity and competencies required for biodiversity management. Moreover, they often feel inadequately trained and have limited opportunities for further training. Furthermore, external contractors may find it challenging to maintain biodiverse gardens effectively.

In general, most professionals involved in iNBIs mentioned they miss meetings with colleagues from other HCFs incorporating iNBIs into their work, hindering broader sharing of insights and best practices, as is offered in communities of practice.

##### 3.3.9.2 Financial obstacles

Maintaining extensive biodiverse vegetation appears to be a challenge, especially in the early years of the project. Additional costs, with the design, implementation, and ongoing maintenance, are often not covered by the patients’ fees, even with funding from, for example, nature conservation organizations, which limits the iNBI continuity. Additionally, there may be delays in allocating budgets to upgrade gardens with amenities such as benches, exercise equipment, additional stairs or wheelchair-accessible paths to improve accessibility, which impacts the improvement and continuity of the iNBI.

##### 3.3.9.3 Infrastructural obstacles

Infrastructure design can significantly impact iNBI implementation. Inappropriate accessibility to the garden can hinder initiatives, particularly when, for example, a psychiatric department is located on a higher floor and access to the garden requires significant time and effort. Interim renovations could temporarily affect access to the natural environment and the iNBI continuity. Furthermore, uneven forest paths pose a challenge for less mobile people and make visits to nature difficult. Although some HCFs have integrated nature into their architecture by incorporating wooden elements and natural photos on the walls, it seems that not enough emphasis is placed on providing easy access to the natural environment. For the integration of iNBI to be seamless, more focus on developing an infrastructure that provides easy access to the outdoors is recommended.

*“The only disadvantage is that we are on the second floor and if the patient cannot leave independently, it is already a ten-minute walk. Yes, the hospital is in a green environment, but it was not built with easy access to the green environment. Yes, now, if a nurse accompanying the patient can be outside quickly, but then the nurse has to come back. We should be located on the ground floor with our department with an open door so that people can go outside easily.” (HCF3 HCP).*

Overall, rather than obstacles, these contextual conditions should be considered to ensure the successful design, implementation and the continuity of the iNBIs.

### 3.4 Toward the fine-tuning of the practical relevance of the quality framework for integrated nature-based interventions

In this section, we first provide a summary of the stakeholders’ assembly review which supported the further choices in the fine-tuning of the practical relevance of the framework.

#### 3.4.1 Brief summary of the stakeholders’ assembly review

The aim of the stakeholders’ assembly was to collect feedback regarding the preliminary framework, resulting from the SCR (32), and to fine-tune the practical relevance of the framework. A report was written from which we summarize the most important insights for this study.

In the absence of an evidence-based iNBI quality framework, the strength of the framework can lie in the fact that it could stimulate the start of an iNBI in an HCF. The participants also mentioned that the framework could serve as a model for innovation and continuous evaluation. Additionally, the framework should be easy to understand, with minimal steps and guiding principles. Because the iNBI is context-bound, the framework should allow flexibility within an adaptive, cyclical process to adapt to changing situations and set priorities. Furthermore, to support the quality and continuity of iNBIs, the participants mentioned that providing cross-boundary education about ecosystems and biodiversity to healthcare staff and the nature-health benefits to NM staff would be beneficial. Another comment regarding its applicability was that the framework targets a single iNBI project. At the same time, it is often linked to concurrent projects, posing a potential threat to the applicability of the framework. Carefully delineating goals and discussing how the iNBI project relates to other projects was recommended to address this. In addition, jointly clarifying terms and terminology, such as ‘biodiversity’ or ‘nature-based intervention’ is essential. As different disciplines were represented in the assembly, varying priorities of each discipline were mentioned. When it comes to collaboration and integration between the NM and healthcare departments in the setting of an iNBI, it was experienced that finding a balance concerning the priorities could be a challenge. In response, a transdisciplinary approach was emphasized to facilitate dialog and shared understanding. Finally, high staff turnover and lack of financial resources were recognized as a risk to commitment to iNBIs and their continuity.

#### 3.4.2 Fine-tuning the quality framework

**Figure 4:**
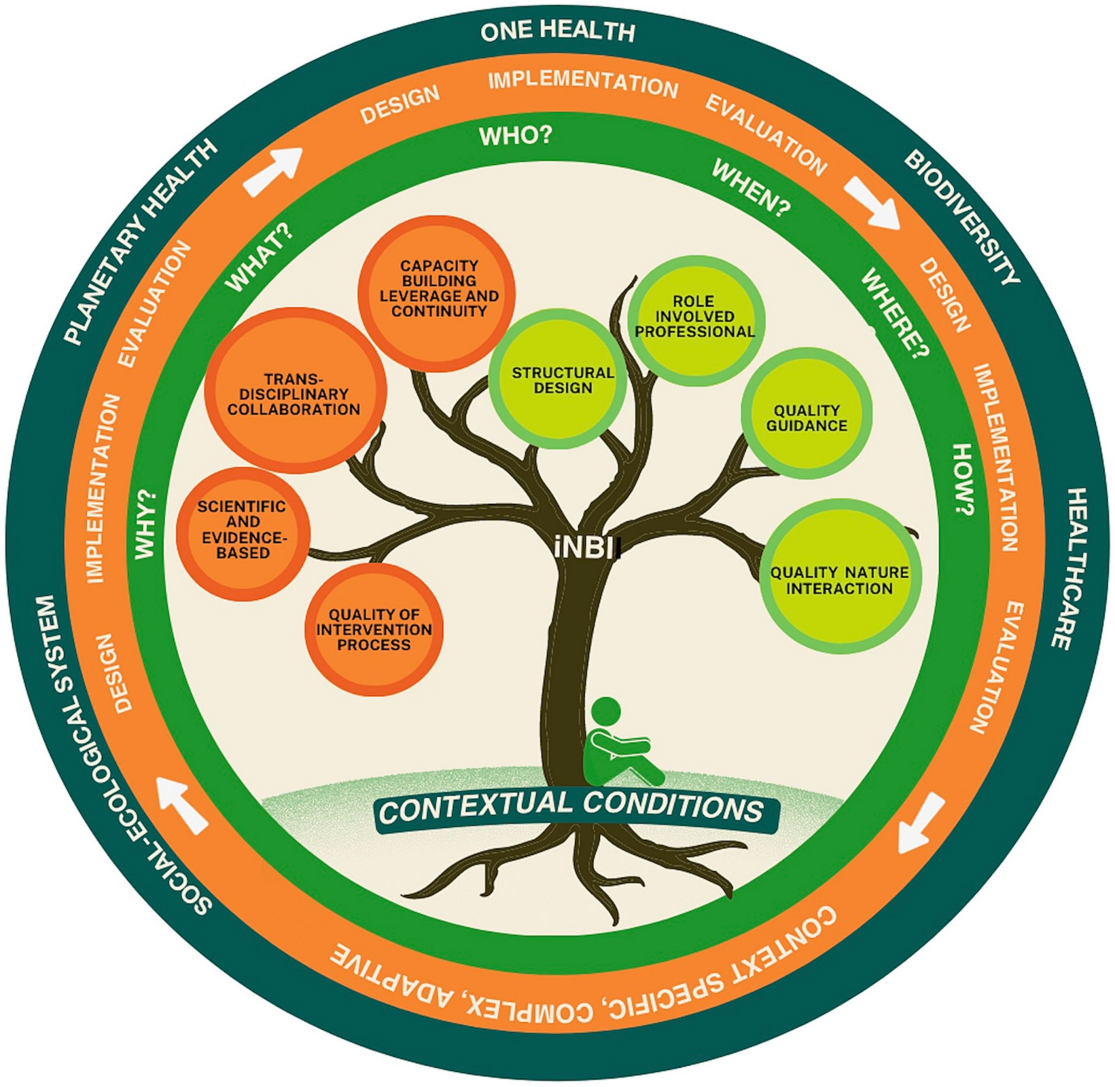
Quality framework for integrated nature-based interventions (QiNBI) in healthcare facilities. The tree represents an ecosystem of quality criteria, all connected to each other, while each have their specific role in the iNBI. Some criteria are logically given greater consideration in the design phase (orange color), and others during implementation (light green color). The contextual conditions at the roots of the tree represent the fundaments for the iNBI. The trunk connects the quality criteria and the contextual conditions, as they cannot be viewed separately. The 5W1H method supports the discussion about the quality criteria at each phase of the process (green circle). The iNBI is characterized by a complex, cyclical, and adaptive process of design, implementation and evaluation (orange circle). The iNBI is embedded in its wider context (dark green circle).

Taken all together, based on 3.2., 3.3. and 3.4.1., combined with discussions with the project steering group, the preliminary framework resulting from the SCR (32), will be fine-tuned on its practical relevance. This results into a new and more complete representation of the quality framework for iNBIs (QiNBI) (Figure 4). In this light, several new components have been added. Below we discuss in detail how the new compiled framework, consisting of four layers, should be understood.

First, the set of quality criteria is represented by the metaphor of a tree, representing the criteria.

The practical relevance of all quality criteria found in the SCR (32), consisting of head criteria with their respective sub-criteria have been confirmed by the respondents. For example, the head criterion ‘Needs analysis’ is supported by the sub-criteria ‘iNBI in general’, ‘users and outdoor environment analysis’, ‘goal setting’ and ‘process and impact evaluation’. In addition, one newly identified head criterion was added, namely ‘Capacity building, leverage and continuity’. Some refinements have been made based on the interviews. For example, compared to the SCR (32), the head criterion ‘evaluation’ has been added as a sub-criterion to each head criterion (see Table 3), since each criterion should be evaluated in each phase. Next, ‘use of theoretical frameworks and scientific evidence’ moved from a sub-criterion to a head criterion level. At the same time, its name was also refined to ‘use of evidence-based frameworks and practices’. Another example of refinement is more at the content level, such as ‘goal setting’, which was added as a sub-criterion to the head criterion of ‘intervention process’. For others, the criterion title was refined (e.g., changing ‘multidisciplinary collaboration’ into ‘transdisciplinary collaboration’, ‘barriers’ into ‘contextual conditions’). Regarding the head criterion ‘role of the professional’ more detail has been gained about the sub-criteria ‘iNBI competencies’ and ‘personal relationship with nature’. Furthermore, the head criterion ‘quality of the interactions with nature’ has been more detailed compared to the SCR (32). However, further research is needed to establish qualities of nature interactions in compliance with the capabilities and needs of a specific target group. The choices made in these refinements were based on what was found in the practice of the HCFs, and on discussions with the project steering group. However, these choices can be criticized, requiring subsequent field-based research to fine-tune these quality criteria and the framework further.

**Table 3:** Example of one head criterion (orange) with one sub-criterion (light gray) of the checklist to operationalize the iNBI quality framework, Process and impact evaluation (dark gray) are repeated after each criterion.

QUALITY CRITERIA BIODIVERSITY/NBI/BIODIVERSITY + NBI (iNBI)
INTERVENTION PROCESS
NEEDS ANALYSIS: iNBI general, users and outdoor environment
The iNBI in general
Why do we want to design the iNBI? (e.g., integrate the biodiversity-health link)
What is the concept of the iNBI in our facility? (e.g., designing a new piece of land with biodiversity where patients of department x can benefit from it)
Who will be the coordinator of this iNBI? (e.g., a specific person, a manager)
Who else should be involved for each domain, in each phase? (e.g., interested HCPs of departments of psychiatry, geriatry, coordinator nature management)
Where is the location of the iNBI we are talking about? (e.g., specific piece of land, or the entire surrounding natural environment)
What do we need to know from each domain? (e.g., needs of guiding HCPs, preferences and risks patients, ecological info)
Users analysis
What are the needs and the barriers of your target group? (e.g., health benefits of being in nature, safety, accessibility)
How will nature support health outcomes (check in the evidence-based literature), and on what will you focus? (e.g., positive effect of biodiversity on mental health and microbiome)
How do we handle contradictory needs versus design components? (e.g., discuss with NM important)
Which instruments, methods will we use to do needs analysis? (e.g., questionnaires, interviews, focus group)
When will we do the analysis? (e.g., in month X-Y)
Who should be involved in the needs analysis (e.g., departments, patients, staff, experts)
Outdoor environment analysis
What type of nature is present and should be designed? What are the risks and how to cover them (e.g., structural design, type of guidance)?
Which instruments, methods will we use to do needs analysis? (e.g., expert elicitation, consultancy, questionnaire)
When will we do the analysis? (e.g., in month X-Y)
Who should be involved in the needs analysis (e.g., departments, patients, staff, experts)
PROCESS AND IMPACT EVALUATION
Is a needs analysis carried out?
Is there a main responsible, coordinator for this iNBI? Who is responble for what?
Is there a transdisciplinary project group?
Are coherent goals formulated for each domain?
Were the goals formulated within a transdisciplinary process?
Are the goals and the progress monitored?
Does the needs analysis have offered the information we were looking for?
What can we do better next time in the needs analysis?
Are the measurement tools used to evaluate evolution of the biodiversity and the health impact of the iNBI adapted to its issues and context, and are they scientific validated?

Some criteria are logically given greater consideration in the design phase (orange colored), while others are emphasized during implementation phase (light green colored). However, in practice, all quality criteria turned out to be relevant for each intervention phase, depending on the context and conditions. Their priority should be considered by the HCF in each phase. In principle, all quality criteria apply for both biodiversity and healthcare, as their integration. However, some criteria may be specific to healthcare, such as ‘Quality of the guidance’. Nevertheless, this criterion needs to be discussed with the NM department, with the view to integrating biodiversity, and vice versa. For example, the type of guidance tailored to the target group can be discussed. In turn, consideration could be given to what this means for the inclusion of biodiverse vegetation in the area where the HCP would guide their target group. Another example could be discussing the health risks of certain vegetation (e.g., insect bites, pollen allergies). Questions to facilitate this dialog should be asked by both sides. For example, how can the biodiverse environment meet the needs of the target group, and how can the goals of biodiversity be met at the same time? Another example is the structural design: what is important to improve accessibility to the biodiverse environment, without contradicting with biodiversity goals? However, the interdependency between the quality criteria will quickly become clear. For example, discussion of the needs will feed the discussion on structural design and capacity building. However, in order not to miss any details, and to manage the complexity of these interdependencies, it is essential to discuss each criterion individually. This involves asking questions and making decisions about how it will be established taking into account the contextual conditions and the development stage of the iNBI. Here, insights can be gained and further deepened as to the extent and at what level (e.g., staff, patients, management) the criteria relate to one another in a specific context.

Second, we added the 5W1H method (what, why, who, when, where, how) to operationalize the tree of quality criteria. To support this process, we developed an example of a checklist (Table 3 - full version in Supplementary material) with questions. The checklist comprises all the quality criteria discussed previously. Using the 5W1H method, each criterion can be discussed individually for each domain and its integration where feasible. An example is given in Table 3. The proposed questions per criterion do not claim to be complete and can be adapted to the context.

Third, the realization of the iNBI is guided by an adaptive design, implementation, and evaluation cycle that allows for dealing with complexity and the specific context. Once the implementation phase is reached, it is advisable to evaluate the criteria considered in the design phase. In addition, the evaluation can also take place during the design and implementation of the iNBI, and depending on the evaluation needs. This supports the continuous monitoring, adjustment, learning and improvement of the iNBI. In the checklist this is achieved by inserting the evaluation criterion after each other criterion. Lessons learned should be immediately integrated or collected to contribute to knowledge creation about iNBIs in the HCF.

Fourth, the iNBI, in which biodiversity and healthcare come together, is embedded in a larger context of planetary concerns (e.g., climate change). Therefore, Planetary and One Health approaches can be helpful to support the quality of iNBIs. They cover the transdisciplinary approach, develop a reciprocal human-nature-health relationship where feasible, and work with complexity. However, it is important to note that, as an iNBI is a context-specific complex, adaptive intervention, it will evolve over time. For example, contextual conditions such as climate change, economic situations, staff turnover, personal-related aspects, or the progress of biodiversity restoration, will always require rethinking of the decisions made regarding the iNBI. This phenomenon is typical for complex interventions (37). The same therefore applies to the framework, which should be understood as something that is evolving and needs to be evaluated and adapted after a few years, due to changing contextual conditions (e.g., climate, context of healthcare) as well as new scientific, practice-based and local knowledge.

## 4 Discussion

Our study led to the fine-tuning of the practical relevance of the quality framework we developed previously (32). To our knowledge, this is the first quality framework for iNBIs taking place in the healthcare organizational setting. Other examples of frameworks can be found in ‘social green prescribing’ (44), a recent national project in England and in ‘green prescriptions’ (45–48), increasingly getting attention in several countries. However, green prescribing and social green prescribing primarily focus on individual and community health, with third-sector organizations providing NBIs. In contrast, iNBIs focus on context-specific, place-based interventions with an emphasis on health promotion strategies that serve simultaneously biodiversity restoration and human health. In addition, the guidance in nature is primarily provided by HCP staff of the respective HCF. Nevertheless, several criteria revealed in our study were also found in these approaches. For example, both apply a person-centered care perspective in the need analysis of the target group (44, 49, 50). Next, transdisciplinary collaborations are established in which co-design is advanced, as well ‘working evidence-based’, and the value of ‘building capacity’ (44, 50). In addition, although still not common, a plea for adding the perspective of Planetary Health can also be found (46). Furthermore, the same challenges are encountered, such as budget constraints, lack of time and human resources to maintain the NBI (44).

It is still too early to provide standardized guidelines for iNBIs to ensure their quality. In addition, the context-specificity, complexity, the person-centered approach leading to a variety of iNBIs, often taking place within unpredictable situations and changing contexts, makes comparing iNBIs and establishing best practices challenging (51). However, the stakeholders’ assembly review and the interviews indicated that the practical relevance of the framework can be helpful to structure the iNBI design, implementation and evaluation.

The quality criteria identified in this study appear to be interacting elements in complex settings. For example, when considering one criterion, it becomes quickly clear that another is involved, as discussed in 3.4.2. Therefore, to address quality in the complexity in iNBIs, a context-specific, adaptive and iterative approach is required (34). Embracing approaches such as One Health and Planetary Health, and implementation science frameworks (e.g., complex interventions, intervention mapping, implementation science) could be helpful in effectively addressing the context-specific, complex, and adaptive nature of iNBIs. In addition, these approaches also show the need for transdisciplinary collaboration. This collaboration takes into account the importance of creating a common language and terminology and understanding, while including patients’ perspectives. These efforts aim to bridge the gaps between the different stakeholders involved (4, 52). Furthermore, our study shows that systematic monitoring and evaluation of the intervention process and iNBI impacts on biodiversity and health remains challenging. The One Health approach could be helpful. This approach promotes continuous evaluation of ‘the sustainable balance of the health of people, animals, and ecosystems. In the meantime the interdependency and close links between the health of humans, domestic and wild animals, plants, and the wider environment (including ecosystems) are recognized’ (4). Therefore, evaluation should happen at different levels of the intervention, from the individual to the societal level.

### 4.1 Implications for practice

The findings of this study have implications for the HCFs, policymakers and education.

#### 4.1.1 For healthcare facilities

Firstly, the framework can serve as a tool for HCFs to structure the iNBI design, implementation and evaluation as it opens the dialog between two different domains (nature management and healthcare), with each their needs and challenges. During the implementation phase the framework and the checklist may serve as a guide. Secondly, it supports the HCFs to work evidence-based, as it encourages reliance on scientific frameworks and evidence-based practices, which has increasing attention in the last decade in Belgium.

#### 4.1.2 For policymakers

At a higher level, healthcare policymakers could encourage HCFs to use the framework and checklist when designing the iNBI, and when applying for funding for an iNBI project. Additionally, governmental funding organizations could coach and support the HCFs during the different iNBI phases based on regular evaluations using this checklist and provide them with recommendations and proactive advice. Furthermore, in Belgium, the Flemish governmental agency responsible for healthcare quality could consider integrating the quality assessment of iNBIs into its current quality healthcare system and encourage further research.

#### 4.1.3 For education in healthcare and nature management

An ‘iNBI-training program’ could be offered to students and professionals in healthcare and NM, to support their role in the iNBI. Topics that could be included are, for example, working with the iNBI quality framework and checklist, Planetary Health Education (53, 54) and the One Health approach (4) including One Health competencies (55) (e.g., effective communication, learning to work transdisciplinary, reflexivity, understanding One Health concepts, harnessing complexity and uncertainty). In addition, teaching about implementation science frameworks (34, 37), complexity science and systems thinking used in the healthcare context (56–58) could also be valuable. Educating HCPs about the specificity of nature interaction quality, intensity, duration, length and frequency (32) and the risk factors that biodiversity can pose for health (e.g., allergic reactions to insect bites, pollen allergies, potential psychological harm related to fear) (30, 59) should also be considered. Furthermore, developing “nature-connected care awareness” (60) by the involved professionals in iNBI, NM and medical students could provide them with insights into their relationship with nature, which is an advantage in the transition to sustainability (2, 7) and being a role model (60). Finally, being trained in knowledge of ecosystems, could contribute to increased awareness of the interdependency of the health of ecosystems, biodiversity and humans (55).

### 4.2 Strengths and limitations of the study

Our study included different types of HCFs in Flanders (Belgium) with different target groups to fine-tuning the practical relevance of the iNBI framework. The diversity in HCFs enabled the development of a framework that includes the context-specificity and complexity of iNBIs.

Given the qualitative nature of this study, various strategies were applied to ensure validity and reliability. First, regular reflexive sessions (61) were held between the PIs to refine interview techniques, data coding, and finalize the findings. Second, member-checking was performed by validating the study findings with the respondents of the interviews. The short timing in which the member check (due to time constraints in the project) had to take place may have been an obstacle regarding availability of respondents. Nevertheless, the answers confirmed the findings and did not require further additions or changes. Third, insights and feedback were given during the stakeholders’ assembly review. Finally, regular meetings with the project steering group were organized to review the findings. Here also, a reflexive stance was adopted, considering the possible bias of the funders and researchers.

Due to time and resources constraints and practical reasons the patient’s perspective has not been taken into account in this study. Their feedback, which is crucial to assessing the effectiveness and relevance of the iNBI, could have led to further refinement of the quality criteria identified in this study or to the identification of additional quality criteria.

Finally, although we have proposed a checklist that can be used for various purposes, its applicability in practice should be further tested on a larger scale in different geographical or socio-economic contexts.

Despite its limitations, this study resulted in a fine-tuning of the practical relevance of the quality framework for iNBIs in HCFs, and its operationalization through a checklist. Since iNBIs are a novel type of intervention, we believe that this study has made a significant contribution to the quality assessment of iNBIs. The framework and checklist can support HCFs in a context-specific, structured, transdisciplinary approach to co-design, implementation and evaluation of iNBIs and inform policymakers and education of students and professionals in healthcare and NM.

### 4.3 Recommendations for research and further work

We recognize that it is not always easy to implement the mentioned frameworks and approaches (e.g., One Health, implementation science, nature-connected care) into professional practice. Consequently, our proposed QiNBI framework and checklist could be considered as a living document and a conceptual contribution, that requires further elaboration and field testing.

The QiNBI framework and checklist can be used as a dialog tool between stakeholders in an HCF, between HCFs or in communities of practice. For example, a subsequent study in Belgium will use the checklist as a dialog tool within and across HCFs, with the aim to develop tools and measurement instruments for the design, implementation and evaluation of iNBIs. Future work could also focus on translating quality criteria into quality indicators with quantitative measures where appropriate. Hereby the complexity and specific context of iNBIs should be considered. Furthermore, the patients’ perspectives should also be included in future research to ensure that interventions are truly patient-centered and effective in improving health outcomes.

As this framework was developed in the Belgian context, further research could explore its applicability on a larger scale for diverse patient populations, in different types of healthcare facilities, geographical areas and socio-economic contexts. In addition, the use of existing healthcare quality frameworks for the design, implementation and evaluation of iNBIs should be examined for its applicability in the respective context, socio-cultural situation and politics.

## Data availability statement

The datasets presented in this article are not readily available because of ethical and privacy restrictions. Requests to access the datasets should be directed to HK, hans.keune@uantwerpen.be.

## Ethics statement

The studies involving humans were approved by the Ethical Committee of the Universal Hospital Antwerp. The studies were conducted in accordance with the local legislation and institutional requirements. The participants provided their written informed consent to participate in this study.

## Author contributions

AS: Writing – original draft, Validation, Methodology, Investigation, Conceptualization. BD: Writing – review & editing, Validation, Methodology, Investigation, Conceptualization. GB: Writing – review & editing, Validation, Investigation. IS: Writing – review & editing. RS: Writing – review & editing. KB: Writing – review & editing. RR: Writing – review & editing, Supervision, Methodology, Conceptualization. HK: Writing – review & editing, Supervision, Methodology, Funding acquisition, Conceptualization.

## Footnotes

^1^ https://www.who.int/groups/one-health-high-level-expert-panel

^2^ https://www.planetaryhealthalliance.org/

^3^ _https://www.zorgkwaliteit.be_
