# Supplementary material for: Fine-tuning the practical relevance of a quality framework for integrated nature-based interventions in healthcare facilities. A qualitative interview study
Source: Front Public Health. 2024 Jun 5;12:1379230. doi: 10.3389/fpubh.2024.1379230 (PMC11186510; doi:10.3389/fpubh.2024.1379230)
Supplement: Supplementary file 1 [file Data_Sheet_1.docx]

**Interview guide for the multiple case study of nature-based interventions in healthcare institutions.**

**Interviewers:** AS, BD, GDB

This is an interview guide as a support during the interview. The questions are not posed in this order, but in a responsive way. The interview guide is meant to be indicative. Questions are ordered per main topic and mentions to whom the questions are relevant.

**Start of the interview**

Welcome and put the person at ease. Explain format of the interview and how it will go. Refer to the email conversation or online introductory session.

Ask the participant to tell something about him or herself personally and professionally, to let the person feel at ease. Hand over the informed consent form and provide additional information upon request. Let people fill in and sign the form before starting the interview and the recording.

**Abbreviations:**

NBI= Nature-based intervention. This is the biodiversity project in combination with use for guidance of target group

iNBI= Integrated Nature-based intervention

HCP= Healthcare Professional

**Questions quality of intervention processes**

**(For every person being interviewed – adapt the question to the participant)**

- **Start of the iNBI-project:**
  - What prompted you to start with this project? (Consider also other nature-oriented projects)
  - How did you get started?
  - Are you (as group) supported in that (e.g., by external partners)?
  - How do you see the link with the mission and vision of the healthcare institution?
- **Needs analysis & goalsetting:**
  - Was a needs-analysis conducted? How?
  - How is the iNBI adopted to the personal needs of the target group? To what extent is it tailor-made and how is this done?
  - How and which goals/objectives are set for this project?
  - Which outcomes/changes do they expect with this iNBI, and at which levels (question here individual (staff and/or patients), institutional, professional)?
  - Who was involved in the goal setting?
- **Organizational culture**:
  - To what extent is the iNBI supported by the entire organization, or by which specific department?
  - To what extent is this intervention supported by the healthcare providers, do you see a lot of potential in it?
  - What interventions have been done to create leverage for this project and the further use and care of nature in healthcare practice?
- **Multi-level approach:**
  - Who is involved and at what stages in the project?
  - What was the contribution and what contribution is still being made by those who were/are involved?
  - How is the management involved?
  - How is collaboration between departments and with stakeholders organized
  - How are healthcare professionals involved, for what, and at what moments/phases in the project?
- **Interdisciplinary approach:**
  - Which discplines are involved?
  - Is there a collaboration with other healthcare institutions or other organizations, and for what, at which moment?
  - Is there a care team that supports the entire NBI?
- **Scientific support:**
  - What methods were used to design the NBI? Which theoretical frameworks underpin it?
  - At what level is there a collaboration with a scientific institute for support in the design, implementation and evaluation of the iNBI?
  - Is there (in)explicitly a model of change formulated, and if yes, which one?
- **Role of the participant in the iNBI**
  - How do you communicate about the iNBI to your target group? What do you tell? E.g. for visitors, patients, … For example, we do not work with pesticides. How do they communicate about it? Or ‘we use this green environment for …’ (adjust the question to the participant)
  - How are you trained in the knowledge or needed competencies in this project?
  - How were you informed and trained regarding design, implementation, use of the iNBI?
- **Sustainability/continuity of the iNBI**
  - How robust is the set-up and further follow-up of the green project, how is it organized?
  - How is the progress of the biodiversity/ecological quality or human health or intervention processes monitored, and adjusted when necessary? Which tools are used?
- **Hindrances, barriers**
  - Which barriers or hindrances do/did they encounter?
  - How were they handled, solved?
  - Do you feel hindered by some formal framework (eg permits, too limited, cooperation with, leave of people, lack of resources …).
- **Evaluation of the effects and progress**
  - What progress do you expect regarding the project in the near future, how do you maintain the project (self or dependent as organization)?
  - How is the progress of the project monitored, and adjusted? How frequently? Which tools are used?

Is there anything else you think is important to share?

**Questions ecological quality**

**(For interview with representative of ecological quality)**

- **Vision on ecological quality:**
  - How would you describe the iNBI-project, garden, natural environment, what was it like before and what are your objectives (in terms of green and blue)?
  - How did you come to those choices, on what theoretical frameworks is the design based? How does this affect your design, your work, choices, freedom to do something?
  - How does motivation or sensibilization of the healthcare professionals regarding this project happen to integrate it into their practice? (for example, is there communication between the green and the healthcare department about the vision on ecology-health topic? What is the communication about?). How do you communicate on the importance of biodiversity to the HCP?
- **Design iNBI-project:**
  - What is the surface of the green project (or surrounding natural environment that can be used for restoring biodiversity and guidance of people )
  - Why did you choose for this type of plants, proportions in the structures (e.g.lawns, trees)
  - How is it managed? Intensity and type of management and maintenance.
  - To what extent does the garden fit in with the wider environment, how have you been inspired by the environment. Is it a reinforcement of the ecosystem outside the institutions (more in a network idea)?
  - Is there a connection with the neighbourhood?
  - Are there any collaborations with external organizations for the design and ecological quality?

Is there anything else you think is important to share?

**Questions quality of human health**

**(For interview with HCP and a management/leader of the organization)**

- **The health intervention in nature, guidance (by whom, what, why, how, when, for whom?) (For interview with HCP and manager):**
  - How would you describe the iNBI we are talking about now?
  - Which vision on health is followed? How is nature part of that??
  - Who guides in nature, and what is their training/ competencies?
  - Who is the target group, what is the purpose, expected outcome?
  - What theoretical frameworks or theory of change are used in the nature-based health intervention?
  - What is the type of guidance in the intervention, and what is its intensity (low, intensive)? Is there reflection included, and on what?
  - How is the transfer of the learnings of the guidance guaranteed to daily life?
  - Which components of nature experience are stimulated (e.g., sensory stimulation, silence, being alone, feeling of getting away,…)
  - How does the design of the green project, and the types of nature responds to the different moods and preferences of the target group? (e.g. research says that preference of certain type of nature is very personal and depending on the mood of the person on a specific moment)
  - How long is the intervention in nature?
  - What are the qualities of nature that are focused on (use of scheme)? How are they reached? Which activities are done?

**The role of the health professional (For interview with HCP)**

- - What is your personal motivation to go into nature with the target group?
  - How would you describe your relationship with nature (interviewer can use scheme to discuss)
  - How do you integrate this into the health practice?
  - What is your approach in this NBI?
  - Which training did you follow to work with people in nature? Is there intervision/supervision?
  - How do you communicate about this NBI to your target group?
  - Which barriers/hindrances do you encounter here?

**Evaluation of the effects (For interview with HCP and manager)**

- - How is the progress of the target group monitored, and adjusted? How frequently?

Is there anything else you think is important to share?
