# Supplementary material for: Fine-tuning the practical relevance of a quality framework for integrated nature-based interventions in healthcare facilities. A qualitative interview study
Source: Front Public Health. 2024 Jun 5;12:1379230. doi: 10.3389/fpubh.2024.1379230 (PMC11186510; doi:10.3389/fpubh.2024.1379230)
Supplement: Supplementary file 2 [file Data_Sheet_2.docx]

**Checklist findings of interviews: quality framework integrated nature-based interventions**

This questionnaire is addressed to the participants of the study 'quality framework for integrated nature-based interventions in healthcare facilities'. With this questionnaire we want to check how well the findings of our analysis match your experience in practice.

Nothing from this questionnaire may be used or reproduced without prior written permission from the authors.

The data generated from this questionnaire is only accessible to the primary researchers, anonymous processing is included in the analysis.

Thank you for your time, involvement and insights.

1. Name

2. Email

3. Organisation

4. Function

5. The figure below gives an overview of the quality framework encompassing all the themes resulting from the analysis of the interviews. The questions below clarify each theme after which you can indicate to what extent the theme in question is recognisable in your practice or your organisation.


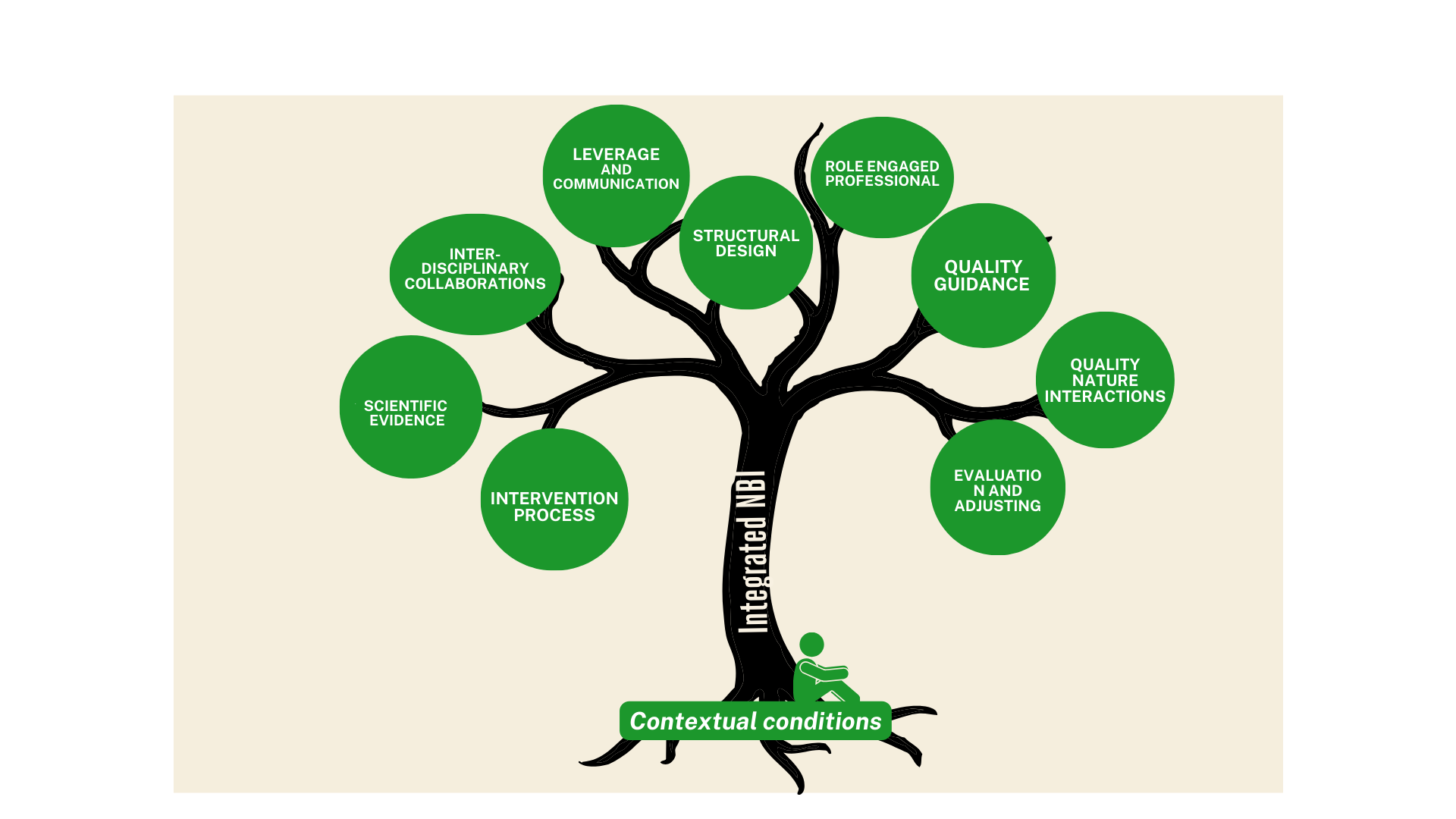


**6. Core elements of intervention - needs analysis 1/4**

The needs analysis for the design and implementation of the care nature intervention is usually done in your organisation through consultation, without the use of a specific system or method.

yes

no

other: clarify

**7. Core elements of intervention - needs analysis 2/4**

When designing the care nature intervention in your organisation, the needs analysis focuses on

the possibilities of person-centred care in the care nature intervention

on the restoration of biodiversity around the care facility

other: clarify

**8. Core elements intervention - needs analysis 3/4**

The needs for the care aspect are mainly detected in your organisation for the care nature intervention

based on own experiences and accumulated knowledge from previous care nature interventions

on the basis of expertise from an external expert

based on the expertise of an internal expert

other: clarify

**9. Core elements intervention - needs analysis 4/4**

The needs for biodiversity restoration around your care facility are mainly detected

based on own experiences and accumulated knowledge from previous care nature interventions

based on expertise from an external expert

based on the expertise of an internal expert

other: clarify

**10. Core elements of intervention - Interdisciplinary collaboration**

The design of a care nature intervention usually takes e interdisciplinary approach. This involves the following parties:

not applicable

other departments (e.g. technical department, care department, HR department)

external experts from the municipality

external experts from the Flemish government

nature or social associations

other: clarify

**11. Core elements intervention - Goals of the care nature intervention**

The goals of the care nature interventions in your organisation mainly include:

supporting the care of patients/clients and employees

supporting preventive care of visitors

restoring biodiversity

sensory stimulation

stimulating interaction between the healthcare facility and the neighbourhood.

other: clarify

**12. Scientific evidence and frameworks**

When designing and during the implementation of a natur- based intervention in my organisation:

we conduct an exploratory scientific literature review

we collaborate with scientific institutions (e.g. university, college, internal research cell, ...) for research on existing care nature interventions in our organisation.

the practical feasibility of science-based methodologies or insights important and dependent on the context of in which the care nature intervention is implemented.

other: clarify

**13. Solid external collaborations**

Most nature-based interventions are implemented with the help of various collaborations with internal and external partners. These collaborations are characterised by the expertise or services contributed. In the design and implementation, we collaborate with:

volunteers

other care facilities

the municipality

nature organisations

other: clarify

**14. Leverage and communication 1/3**

In my organisation, regarding the nature-based intervention, there is

sufficient support from my management

sufficient support from the executives/management

sufficient support among employees across departments

sufficient support only among employees in my department

there is a need for more support from the management

there is a need for more support from the different departments

there is a need for more support from my department

other: clarify

**15. Leverage and communication 2/3**

My organisation commits to internal communication about the integrated nature-based intervention

no

yes, to all sections of the organisation

yes, during all phases of the project

other: clarify

**16. Support strategies and communication 3/3**

My organisation engages in external communication about the healthcare nature intervention

no

to visitors

to the neighbourhood

to stakeholders we are/were collaborating with

other: clarify

**17. Structural design: quality of design for care needs and biodiversity needs**

When designing an iNBI, it was found that in our organisation that:

the accessibility aspect has priority, adapted to the specificity of the target group.

the use of structural elements is important (structural elements are: benches, specific spots (wonder, comfort spot, ...), sound elements, paths, the presence of animals (e.g. chickens, donkeys, dog)).

the aspect of biodiversity is important in structural design

in order to improve the quality of biodiversity, the use of local planting and appropriate management are the main objectives.

other: clarify

**18. Your role as an engaged professional in the iNBI consists of:**

designing and implementing a care nature intervention requires additional competences. Which ones are recognisable to you?

having sufficient flexibility to deal with changing and complex circumstances

daring to experiment and learn through experience

providing support in securing the care nature intervention (e.g. also broadening one's own ecological knowledge, training in guiding in nature)

being able to share one's own nature experiences with colleagues and staff.

looking for nature myself (for rest, relaxation, enjoying it, admiring its beauty)

other: clarify

**19. Quality of guidance 1/2**

The quality of guidance is supported by several criteria. Please indicate what is applicable or recognisable in your organisation when designing and implementing an iNBI.

The guidance of our target group in the iNBI is mainly conducted by

occupational therapists

physiotherapists

animators

other: clarify

**20. Quality of guidance 2/2**

We try to adapt the INBI to person-centred care

yes

no

other: clarify

**21. Integration of different types of nature interactions in the iNBI**

Different types of interactions with nature can be envisaged in a care nature intervention. Tick the factors that are important in your organisation for successfully designing and implementing a care nature intervention in your healthcare facility.

doing activities in the natural environment (e.g. walking (with patients), gardening)

stimulating sensorial experience (sensory stimuli, such as stimulating smell, taste, feel, hear)

enjoying and marvelling at the beauty of the natural environment

seeing or using metaphorical images (nature as a mirror or teacher) (e.g. planting seeds and caring for the plant, like caring for oneself)

letting people experience the healing power of nature (sense of timelessness, comfort, unwinding)

letting people experience meaning (e.g. by taking responsibility for something in the garden, caring for something)

focus on deepening one's relationship with nature, or connection with nature

other: clarify

**22. Evaluation and adjustment**

Please indicate what is applicable in your organisation

there is still little systematic evaluation of health effects

the quality of biodiversity is still little evaluated in a systematic way

for the health progress and effects, it is mostly through the follow-up discussion with the patient how the patient experienced the intervention.

for the biodiversity aspect, comparisons are mainly based on what you see changing over the years.

other: clarify

**23. Contextual factors**

Listed below are several contextual factors that function as preconditions of the care-nature intervention. They are also often considered obstacles if they are absent or inadequate. Tick the factors that play a role in care nature intervention in your care facility.

Having:

sufficient financial resources for biodiversity planting and management

sufficient expertise for biodiversity planting and management

sufficient time to enjoy the natural environment of the care facility yourself as a professional

sufficient supervisors to guide our target groups in the natural environment of the care institution

sufficient time within the tasks we already perform to accompany our target groups in the natural environment of the care facility

sufficient time within the tasks we already do to maintain and manage the care nature intervention

other: clarify

**24. Comments - suggestions**

Would you like to add anything else that might be important for the quality of iNBI? Think both along the biodiversity side, guiding the target group in the natural environment, and the use of nature for and by the staff themselves.
