# Supplementary material for: Fine-tuning the practical relevance of a quality framework for integrated nature-based interventions in healthcare facilities. A qualitative interview study
Source: Front Public Health. 2024 Jun 5;12:1379230. doi: 10.3389/fpubh.2024.1379230 (PMC11186510; doi:10.3389/fpubh.2024.1379230)
Supplement: Supplementary file 3 [file Data_Sheet_3.docx]

**QiNBI-checklist: Quality Framework for integrated Nature-based interventions checklist**

The checklist guides designing, implementing, and evaluating a context-specific, complex and adaptive iNBI. Although some criteria receive more attention at a particular phase (orange for design and green for implementation), discussing them in each phase is recommended. Then, set priorities for each domain (biodiversity, healthcare, the integration of both). The 'process and impact evaluation' criterion comes into play for each criterion. It checks the quality of the respective criterion and enables regular monitoring and adjustments. It can be used at any phase and interim evaluation moments, depending on the intention of the evaluation. The proposed questions per criterion do not claim to be complete and can be adapted to the context.

The 5W1H method can be helpful in discussing each criterion, where applicable (guiding questions):

- Why (objective): Why is this important?
- What (concept): What is it about in our case?
- Who (people): Who is involved (multi-level and transdisciplinary approach where possible)
- When: When should it be executed?
- Where (location, or in the practice): Where should it happen?
- How (methods, tools): Which approach, tools or instruments can be to used to design, implement or to evaluate this criterion respectively the domain?

| **QUALITY CRITERIA: BIODIVERSITY/HEALTHCARE/HEALTHCARE & BIODIVERSITY** |
| --- |
| **INTERVENTION PROCESS** |
| **NEEDS ANALYSIS: users and outdoor environment** |
| **The iNBI in general** |
| Why do we want to design the iNBI? (e.g., integrate the biodiversity-health link) |
| What is the concept of the iNBI in our facility? (e.g., designing a new piece of land with biodiversity where patients of department x can benefit from it) |
| Who will be the coordinator of this iNBI? (e.g., a specific person, a manager) |
| Who else should be involved for each domain, in each phase? (e.g., interested HCPs of departments of psychiatry, geriatry, coordinator nature management) |
| Where is the location of the iNBI we are talking about? (e.g., specific piece of land, or the entire surrounding natural environment) |
| What do we need to know from each domain? (e.g., needs of guiding HCPs, preferences of and risks for patients, ecological info) |
|  |
| **Users analysis** |
| What are the needs and the barriers of your target group? (e.g., health benefits of being in nature, safety, accessibility) |
| How will nature support health outcomes (check in the evidence-based literature), and on what will you focus? (e.g., positive effect of biodiversity on mental health and microbiome) |
| How do we handle contradictory needs versus design components? (e.g., discuss with NM important) |
| Which instruments, methods will we use to do needs analysis? (e.g., questionnaires, interviews, focus group) |
| When will we do the analysis? (e.g,. in month X-Y) |
| Who should be involved in the needs analysis (e.g., departments, patients, staff, experts) |
|  |
| **Outdoor environment analysis:** |
| What type of nature is present and should be designed? What are the risks and how to cover them (e.g., structural design, type of guidance)? |
| Which instruments, methods will we use to do needs analysis? (e.g., expert elicitation, consultancy, questionnaire) |
| When will we do the analysis? (e.g., in month X-Y) |
| Who should be involved in the needs analysis (e.g., departments, patients, staff, experts) |
|  |
| **GOAL SETTING** |
| What are the main objectives/goals for each domain? (e.g., biodiversity restoration of a certain piece of land, improving health with complementary approach) |
| Why are they important? (e.g., contribution to sustainable healtcare, Planetary Health, One Health) |
| When should these goals be met for each domain? (e.g., 3 years for biodiversity) |
| Who will be involved to define the goals to ensure a transdisciplinary approach? (e.g., NM coordinator, involved HCPs) |
| How to monitor the progress of the goals? (e.g., interim evaluations and adjustments) |
|  |
| **PROCESS AND IMPACT EVALUATION** |
| Is a needs analysis carried out? |
| Is there a main responsible, coordinator for this iNBI? Who is responble for what? |
| Is there a transdisciplinary project group? |
| Are coherent goals formulated for each domain? |
| Were the goals formulated within a transdisciplinary process? |
| Are the goals and the progress monitored? |
| Does the needs analysis have offered the information we were looking for? |
| What can we do better next time in the needs analysis? |
| Are the measurement tools used to evaluate evolution of the biodiversity and the health impact of the iNBI adapted to its issues and context, and are they scientific validated? |
|  |
| **SCIENTIFIC EVIDENCE AND FRAMEWORKS** |
| **USE OF SCIENTIFIC EVIDENCE (FINDINGS, THEORIES, FRAMEWORKS)** |
| On which scientific evidence can we rely for each domain (e.g. evidence on biodiversity-health; One Health; Planetary Health; Complex interventions; nature-health;nature connectedness)? |
| How to translate them into practice? (e.g., visualizing by schemes and connecting with activities) |
| Who will be in charge to look for this information? (e.g., internal scientist, coordinator, interested HCP) |
| Which nature-health theory and evidence supports the iNBI? (e.g., Attention Restoration, Stress Reduction, Biophilia, biodiversity hypothesis, nature deficit disorder, biodiversity and health, ..). |
| Which evidence-based intervention process framework supports the iNBI process? (e.g., complex interventions, intervention mapping, implementation science, One Health) |
|  |
| **COLLABORATION WITH SCIENTIFIC INSTITUTION** |
| Do we need to work together with a scientific institution, and if yes, for what exactly? (e.g., monitoring biodiversity, health impact, design process) |
| How can the collaboration with a scientific institution contribute to the quality of the iNBI? (e.g., offering scientific approach in monitoring, evaluation, collecting evidence) |
|  |
| **PROCESS AND IMPACT EVALUATION** |
| Is the iNBI based on scientific evidence? |
| Is the process of the iNBI structured by use of theoretical frameworks? |
| Is there a collaboration with a scientific institute, and if yes, what does it consist? |
|  |
| **TRANSDISCIPLINARY APPROACH** |
| **INTERNAL COLLABORATIONS (which disciplines, departments)** |
| Who/which departments/disciplines should be involved per domain (e.g., staff, patient; nature management, psychiatry, ...)? |
| When specifically should they be involved? (e.g., from the beginning, during evaluation moments) |
| Why is the collaboration with each person/department important? (e.g., relying on internal expertise, creating leverage) |
| What will be their role or contribution (e.g., giving input in design, contribution during implementation)? |
| How will we reach them to invite to collaborate? (e.g., interna meeting, email, internal newspaper, personal invitation) |
| Do we have enough internal expertise? (e.g., ecological professional trained in biodiversity, HCP trained in nature therapy) |
|  |
| **EXTERNAL COLLABORATIONS**  (e.g., volunteers, professional partners, healthcare associations, other stakeholders, ...): |
| Do we need external expertise, and for what? (e.g., ecological design garden, biodiversity, integrating planetary health) |
| What will be their role or contribution (e.g., giving input in design, contribution during implementation, funding)? |
| Why is the collaboration with the external partner important? (e.g., capacity building, involvement stakeholders, combining strenghts, honouring networks and locality) |
|  |
| **PROCESS AND IMPACT EVALUATION** |
| Is there a structural collaborationn with internal and external partners? |
| What is the quality of these collaborations? What is their specific contribution and role in the iNBI? |
| Is the quality of the expertise monitored and evaluated and how? |
| How is the quality of external collaborations monitored and evaluated? |
|  |
| **CAPACITY BUILDING, LEVERAGE, CONTINUITY** |
| **INTEGRATION IN VISION** |
| How will the iNBI be integrated in the vision and policies of the healthcare department? (e.g., integrating in our written vision document and communicating to staff) |
| How will the iNBI be integrated in the vision and policies of the nature management department? (e.g., integrating in our written vision document and communicating to staff) |
| How will iNBI be integrated in general company vision? (e.g., integrating in our written vision and strategic) |
|  |
| **KNOWLEDGE RETENTION** |
| How will be iNBI knowledge, experiences and expertise be stored, managed? (e.g., reports, iNBI roadmap, documentation, photos) |
| How will be iNBI knowledge, experiences and expertise be retained? (e.g., creating leverage for involvement, pro-active mentoring of potential iNBI professionals) |
| How to be involved in a community of practice? (e.g., checking of the existence of such network, starting ourselves) |
|  |
| **LEVEL OF INVOLVEMENT OF STAFF** |
| Who will be involved? (e.g. needs analysis, implementation, evaluation) |
| What exactly is their role and what are the tasks? (e.g., inform, decision-making, be role model) |
|  |
| **LEVEL OF INVOLVEMENT OF MANAGEMENT** |
| Who will be involved? (e.g., needs analysis, implementation, evaluation) |
| What exactly is their role and what are the tasks?(e.g., inform, decision-making, being role model) |
|  |
| **GENERATING IDEAS BY STAFF** |
| How will we stimulate the generation of ideas? (e.g., asking, sponsoring ideas) |
| How to collect them? ((e.g., structured moment in meetings, digital idea box, world cafe) |
| Who will be responsible to follow up the ideas? (e.g., someone specific, manager, iNBI professional) |
| For what purpose? (e.g., involve people, getting recognition for iNBI) |
| When? (e.g., monthly, at certain events) |
|  |
| **USE OF NATURAL ENVIRONMENT BY STAFF** |
| Will the staff also use the biodiverse environment for personal use? |
| Why will it be used? (e.g., relaxing, team meetings, pauses) |
| When will it be used (e.g. during pauses, during work, after work) |
|  |
| **FEEDBACK** |
| About what do we want feedback? (e.g. experience of the iNBI) |
| Who should give feedback? (e.g., patients, family, interns) |
| What type of feedback? (e.g., oral, written) |
| Who will collect the feedback? (e.g., HCPs, someone appointed) |
| How to collect the feedback (e.g., listening by HCP, conversations) |
| How will the collected feedback be shared with the team/management? (e.g., report, meeting) |
|  |
| **INTERNAL COMMUNICATION** |
| What to communicate? (e.g. progress biodiversity, departments guiding patients in the biodiverse garden, initiatives concerning the iNBI) |
| Why to communicate? (e.g., informing, sensibilising, honouring, stimulating participation) |
| What is our target group? (e.g., other staff, patients) |
| How to work on the perception of new biodiverse projects? (e.g. explaining about evolution of biodiversity, its value) |
| How to communicate? (e.g., digital, flyers) |
| How to monitor the impact of the communication? (e.g, questionnaire, conversations) |
|  |
| **EXTERNAL COMMUNICATION** |
| What is the pupose of the external communication (e.g., creating leverage externally, role modeling in the healtcare sector) |
| What will be communicated about the iNBI? (e.g., evolution iNBI project, examples of nature interactions in practice) |
| Who is the communication adressed to? (e.g., family, visitors, local community, municipality, professional partners) |
| How to work on the perception of new biodiverse projects? (e.g., communication boards, zoning strategies, signposts) |
| How will be communicatied? (e.g., report, website, social media) |
| When will be communicated? (e.g., trimesterial, weekly) |
| How to monitor the impact of the communication? (e.g, questionnaire, conversations) |
|  |
| **PROCESS AND IMPACT EVALUATION** |
| Is there a commitment to providing information for various target groups (patients, doctors, visitors, etc.) |
| Is there a coherent vision regarding the iNBI? |
| Is active involvement of employees from all levels of the organisation encouraged and honoured? |
| Is there a clear communication strategy and plan? |
| How is the quality and the impact of the communication monitored? |
| Are there strategies for knowledge management and retention? |
|  |
| **STRUCTURAL DESIGN** |
| **SCALE AND DESIGN** |
| What is the scale of the land that will become biodiverse? |
| What is the potential for biodiversity? (e.g., ecological condition) |
| What vegetation can be planted? (e.g., ecological condition, needs target group) |
| Which maintenance is needed and at what frequency? (e.g., weekly, monthly) |
| What will be the design of the biodiverse land/which elements? (e.g., shrubs, trees, ponds, beehive) |
| Who will help and be involved with the design? (e.g., external expert) |
|  |
| **ACCESSIBILITY** |
| Where is the need to create accessibility? (e.g. pathway between HCF and biodiverse garden) |
| What are the needs? (e.g., wheelchair-friendly walkways, raised plant beds) |
| Who is responsible for this? (e.g., technical service, external partner) |
| For who is the biodiverse environment accessible? (e.g., patients, staff, visitors, local community) |
| How is safety secured? (e.g., fences around ponds, no poisonous and prickly plants) |
|  |
| **USE OF SUSTAINABLE MATERIALS** |
| What materials will be used? (e.g., recycled, ecological) |
| Who will monitor this? |
|  |
| **USE OF STRUCTURAL ELEMENTS** |
| Which structural elements will be used to stimulate participation in an iNBI ? (e.g., barefoot paths, benches, exercise equipment, playgrounds, and auditory elements) |
| Which structural elements for biodiversity will be used? (e.g., pond, beehive, herbeceous vegetation, nesting boxes) |
| Which structural elements in the design of the outdoor environment are needed for accessibility, inclusion, sustainability, biodiversity restoration? (e.g., fences, wheelchair paths, beehive) |
|  |
| **PROCESS AND IMPACT EVALUATION** |
| Is sufficient attention paid to the structural elements of the natural environment and the pieces that will be biodiverse? |
| Is accessibility ensured for who it is needed? |
| Are the structural elements in sustainable material? |
|  |
| **ROLE OF THE INVOLVED PROFESSIONALS** |
| **SPECIFIC INBI COMPETENCIES**  (e.g., flexibility, learning by experience, self-disclosure, ecological awareness, One Health competencies, ...) |
| What specific competencies and attitudes towards nature of the healthcare professionals are needed to guide the target group in the outdoor environment? (e.g. ecological knowledge, qualities of interactions with nature, person-centred care) |
| What training is necessary? (e.g. One health competencies, nature-connected care awareness, planetary health education) |
|  |
| **PERSONAL RELATIONSHIP WITH NATURE** |
| What is the level of the relationship with nature of the iNBI professional? (e.g., jogging in nature, deeply nature connected) |
|  |
| **PROCESS AND IMPACT EVALUATION** |
| Is sufficient attention paid to the specific competences needed by iNBI professionals in establishing, securing and supervising an iNBI? |
| Are the competencies monitored and evaluated, and when? |
| Is there training provided where necessary? |
|  |
| **QUALITY OF THE GUIDANCE** |
| **GUIDANCE OF TARGET GROUP** |
| Who will guide in the iNBI? (e.g., occupational therapist, physiotherapist, psychologist, animator) |
| How much guidance (intensity) is needed in the interactions with nature? (e.g., target needs continuously guidance, or can work alone in the garden with instructions) |
| What will be the duration, frequency and lenght of the sessions in nature be? (e.g., 20 minutes three times a week). How long will the NBI-intervention last (e.g., some weeks, some months, …) |
| Is a multidisciplinary healthcare team necessary to support needs and the guidance of the target group in the outdoor environment? Who should be involved? (e.g., HR, other neutral HCPs, internal or external psychologist) |
| Will the guidance be one-on-one or in group? |
| How will safety be secured in the guidance? (e.g. alertness for allergies, psychological or physical barriers) |
|  |
| **PERSON-CENTRED CARE** |
| How will the activivies be tailered to the person? |
| How will the activities be tailored in groupsetting? |
| What is needed to tailor the activities? (e.g., knowledge, user analysis) |
|  |
| **PROCESS AND IMPACT EVALUATION** |
| Are the activities and the guidance person-centred? |
| Are the activities structured bt their intensity, duration and frequency? |
|  |
| **QUALITY OF THE INTERACTIONS WITH NATURE** |
| **ACTIVITIES IN NATURE/GARDENING/SENSORY STIMULATION/AESTHETICAL EXPERIENCE/NATURE AS MIRROR-TEACHER/HEALING POWER/SENSE OF PURPOSE** |
| Which nature interaction will be aimed for? |
| Where will these interactions with nature take place? |
| What will be the contribution of this interaction with nature for the person (desired outcomes)? (e.g., wellbeing, stress reduction, sense of belonging, self-awareness, connection with self, others, nature, increased ecological awareness) |
| How will the interaction be tailored to the target group? (e.g., user analysis, preferences, experiences) |
| Which scientific evidence lies at the base? (e.g, attention restoration theory, biophilia, biodiversity-health) |
| How is worked on deepening nature connectedness and the reciprocal relationship with nature (where applicable)? (e.g., beauty, exercises about giving back to nature) |
| Which potential risks should to be taken into account, and how to handle them? (e.g., allergic reactions to insect bites, pollen allergies, heatstroke) |
| How will the impact of the nature interaction be monitored and evaluated? (e.g, scientific research, interviews, survey) |
|  |
| **PROCESS AND IMPACT EVALUATION** |
| What interactions with nature are envisaged and are they sufficiently tailored to the target audience? On what scientific evidence or frameworks are they based? |
| How is the reciprocity enabled in the different interactions with nature? |
| Are the potential risks listed and are strategies established to effectively respond to them? |
| Are the patients involved in deciding on the offer of interactions with nature? |
| Is the quality of the nature interaction monitored and evaluated and when? |
|  |
| **CONTEXTUAL CONDITIONS** |
| **HUMAN RESOURCES** |
| How many human resources does the iNBI need? |
| How to ensure sufficient staffing for the iNBI? |
| How will be responded to staff shortage to ensure the quality and the continuity of the iNBI? |
|  |
| **FINANCIAL RESOURCES** |
| What are the costs of the iNBI on short term (design), mid-term (implementation) and longterm (maintenance)? |
| What are the opportunities for funding by governmental organisations? |
| For how long is the funding needed? |
| How will the costs be monitored? |
|  |
| **AVAILABILITY (TIME, EMBEDDED IN WORK)** |
| How will the iNBI tasks and guidance be integrated in the daily practice? |
| What is needed to structure the job? |
| What barriers or obstacles can hinder the iNBI continuity? How to anticipate on these barriers? |
|  |
| **CONDITIONS OUTDOOR ENVIRONMENT** |
| What existent structural elements can be a barrier for accessibility? (e.g., paved road) |
| What conditional aspects can be a barrier for restoring biodiversity? (e.g., contaminated soil, scale too small) |
| What actions need to be taken before the start of the iNBI? |
| What future projects can hinder the continuity of the iNBI (e.g. construction project, climate conditions) |
|  |
| **PROCESS AND IMPACT EVALUATION** |
| Were the contextual conditions considered? |
| Are they monitored? |
| Is there attention for the continuity if there are constraints regarding the contextual conditions? |
| How is responded to potential barriers in the outdoor environment? |
|  |
